# When the Matrix Warns: Unraveling Brain Extracellular Matrix Components as Danger Signals in Glioma Progression

**DOI:** 10.3390/ijms27198757

**Published:** 2026-09-30

**Authors:** Anna A. Grechukhina, Tatiana A. Mishchenko, Sergey V. Gudkov, Maria V. Vedunova

**Affiliations:** 1 Institute of Biology and Biomedicine, Lobachevsky State University of Nizhny Novgorod, 23 Gagarin Ave., 603022 Nizhny Novgorod, Russia; anna.vedunova@unn.ru (A.A.G.); saharnova87@mail.ru (T.A.M.); 2 Prokhorov General Physics Institute of the Russian Academy of Sciences, Vavilov Str. 38, 119991 Moscow, Russia; s_makariy@rambler.ru; 3 Department of Fundamental Sciences, Bauman Moscow State Technical University, 5 2nd Baumanskaya Str., 105005 Moscow, Russia

**Keywords:** extracellular matrix, DAMPs, CNS, brain, glioblastoma

## Abstract

The brain constitutes a biologically unique environment tasked with executing the most sophisticated functions of information processing and storage. This singularity arises from several factors, including its status as an immunoprivileged organ. Moreover, the brain extracellular matrix (ECM) provides a structural scaffold for cells and simultaneously acts as an active mediator for information storage and transmission. Unsurprisingly, the ECM has become firmly embedded as a key component of the tetrapartite synapse concept, which has gathered considerable traction in recent decades. In the present review, we aim to characterize the principal ECM components in the context of normal physiology, pathology, and damage-associated molecular patterns (DAMPs). Understanding this perspective is key to neurooncological progression, since the brain’s immune responses remain distinct even when the blood–brain barrier is compromised. A detailed examination of individual ECM constituents offers valuable insight into the mechanisms of tumor-driven microenvironment remodeling. Thus, these molecules should be considered not merely surrogate markers, but rather active drivers of key pathological processes in the central nervous system (CNS). Herein, we summarize and systematize the current evidence on extracellular DAMPs, paying special attention to their relevance in the brain. Our analysis focuses on ECM components that are abundant and functionally significant in the CNS, including the small leucine-rich proteoglycans (decorin, biglycan, and asporin), heparan sulfate proteoglycans, as well as tenascin C, versican, and hyaluronic acid.

## 1. Introduction

Immunogenic cell death (ICD) is currently regarded as a complex mechanism underlying the activation of a targeted immune response during the development of pathological processes at the cellular level. Damage-associated molecular patterns (DAMPs) act as inducers of ICD by activating immune responses through specific receptor-dependent pathways. The classical definition of DAMPs encompasses damage-associated molecules released upon cell death or injury [1]. Subsequently, extracellular DAMPs—molecules that are normally present in the extracellular space as structural components of the extracellular matrix—were characterized. Specific modification or release of individual components of this biopolymer structure can trigger a targeted immune response, allowing these molecules to be classified as non-classical DAMPs. On the other hand, certain individual components of the extracellular matrix are capable of influencing brain function without eliciting activation of the T-cell-dependent immunity; they may likewise be classified as DAMPs, insofar as they arise upon injury or pathology. The appearance of DAMPs in the intercellular space does not necessarily result from cell death but may instead arise from enzymatic degradation of the matrix (e.g., by matrix metalloproteinases (MMPs)) or pathological matrix remodeling [2].

Such molecules signal disruption of tissue structural integrity even before cell death occurs. They serve as early biomarkers, as their presence in the blood or synovial fluid may indicate the onset of degenerative processes, such as osteoarthritis [2] or liver fibrosis [3] at their earliest stages, while cells remain functionally intact [4]. We will endeavour to distinguish between these concepts, separating molecules associated with ICD from molecules that are active participants in other processes within the central nervous system (CNS).

Extracellular DAMPs frequently induce persistent, low-grade sterile inflammation. Because the matrix undergoes continuous turnover and degradation in chronic diseases, such as atherosclerosis, Alzheimer’s disease (AD), and pulmonary fibrosis, these DAMPs remain continuously present in tissues [5]. These molecules, rather than intracellular DAMPs, most frequently sustain the chronic inflammatory environment that leads to fibrosis and loss of organ function [6]. When present outside cells, in the extracellular space and bloodstream, these molecules are more readily neutralized by monoclonal antibodies, synthetic decoy peptides, or enzymes that cleave pathogenic matrix fragments [7].

Extracellular DAMPs do not merely activate toll-like receptors (TLRs); they often carry “hidden” information. For example, in addition to acting as a DAMP, decorin is a natural inhibitor of transforming growth factor-beta (TGF-β) [8]. Therefore, the study of extracellular DAMPs provides insight both into inflammation and into the mechanisms of regeneration, angiogenesis, and cell migration, which are of particular importance in the context of cancer development. Matrix degradation occurs continuously within the tumor microenvironment, and understanding the role of extracellular DAMPs in these processes enables the development of strategies aimed at “reprogramming” matrix signals to enhance cancer immunotherapy [9].

Microglia and astrocytes are not passive bystanders in these processes. Previously, they were regarded as supportive cells that merely “clear debris” or “support” neurons. It is now clear that microglia become active effector of damage upon the release of DAMPs such as biglycan, decorin, high-mobility group box 1 (HMGB1), and mitochondrial DNA through TLR/NLR receptors; they initiate NF-κB-dependent signaling cascades and release tumor necrosis factor alpha (TNF-α), interleukin-1 beta (IL-1β), and reactive oxygen species (ROS), which are directly toxic to synapses and neurons. Thus, inflammation itself becomes a driver of degeneration [10,11].

Unlike many other solid tumors, glioblastoma (GBM) actively utilizes ECM not only as a structural scaffold but also as a source of survival signals [12,13,14]. Investigation of these DAMPs provides insight into how the tumor reprograms its microenvironment. Thus, these molecules function not merely as markers but as drivers of key pathological processes in the brain. In this review, we analyzed and systematized current data on extracellular DAMPs of particular relevance to the brain. The review focuses on ECM components that are abundant and functionally important in the CNS, including decorin, biglycan, asporin, heparan sulfate proteoglycans, as well as tenascin-C versican, and hyaluronic acid.

## 2. Brain Extracellular Matrix Components Involved in CNS Pathological Processes

Brain tissue injury leads to ECM degradation resulting in the release of extracellular DAMPs. This process generates fragments of ECM components which arise through proteolysis mediated by enzymes released from dying cells or by proteases activated during tissue repair and remodeling. In addition, extracellular molecules such as fragments of ECM can function as DAMPs; these components play an important role in inflammatory processes [15].

Soluble DAMPs located in the extracellular space engage with a variety of TLRs, promptly initiating an inflammatory cascade. The structural and chemical profile of these DAMPs is highly variable, encompassing a range from small molecular compounds to large proteins over 100 kDa [16,17]. This inherent structural diversity not only permits these molecules to exhibit broad cross-reactivity with different receptors but also enables them to bind to numerous receptors outside the immune system, thereby significantly amplifying the multifaceted nature of DAMP-driven signaling [18].

### 2.1. Decorin

Decorin is a member of the small proteoglycan family and is synthesized by fibroblasts and myoblasts [8]. It has a molecular mass of 90–140 kDa and consists of a leucine-rich repeat protein core and a glycosaminoglycan chain composed of either chondroitin sulfate or dermatan sulfate [19]. The intact, soluble decorin molecule, with a molecular mass of approximately 100 kDa (a 40 kDa core plus a GAG chain) exhibits DAMP properties. Decorin participates in collagen matrix formation across various tissue types and thereby plays an important role in maintaining tissue structural integrity and function, including in the brain [20].

Decorin, along with other ECM components, has been shown to have significant prognostic value when measured in the cerebrospinal fluid in various neurodegenerative diseases [21]. Elevated decorin levels are associated with the rate of cognitive decline in Alzheimer’s disease [22]. Furthermore, the levels of both decorin and biglycan are increased in multiple sclerosis lesions and areas of demyelination [23,24].

Decorin inhibits the growth of various tumor cell types in vitro and in vivo through multiple mechanisms [25]. For instance, decorin suppresses prostate tumor growth by acting on epidermal growth factor receptor (EGFR) and androgen receptor signaling pathways [26]. Furthermore, in breast cancer, it regulates ErbB family receptors (including EGFR and ErbB2) and attenuates their signaling [27]. In colorectal cancer, decorin induces the translocation of a cyclin-dependent kinase inhibitor p21 into the nucleus, leading to cell cycle arrest in the G1 phase [28]. In the context of GBM, decorin expression inhibits not only the bioactivity of TGF-β but also the expression of TGF-β1 and TGF-β2 mRNA [26,29].

**Figure 1:**
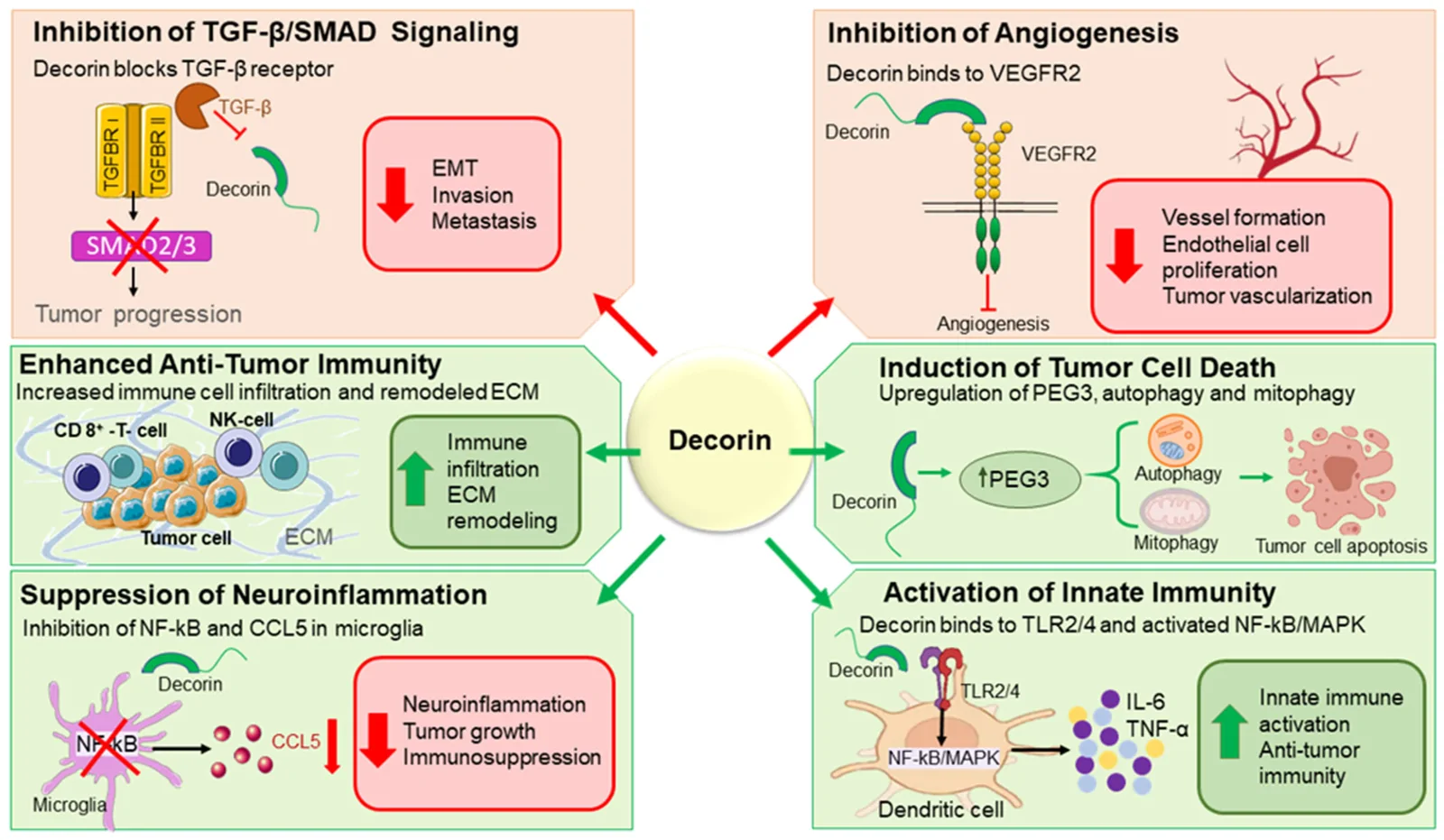
Multiple effects of decorin in the glioblastoma microenvironment. Decorin suppresses glioblastoma progression by inhibiting TGF-β and NF-κB, inducing autophagy, and facilitating T-cell infiltration, while also serving as a marker of anti-VEGF therapy.

Decorin can regulate ECM structure, stabilize blood vessels, suppress angiogenesis, reduce inflammation, and enhance the accessibility of tumor tissue to immune cells [30]. Decorin exerts potent antitumor effects by interfering with TGF-β signaling and activating inflammatory responses through TLR2/4 (Figure 1) [31]. It reduces matrix stiffness and facilitates the infiltration of immune cells, particularly T cells, into the tumor [32]. Decorin also acts on microglia by suppressing the production of the growth factor CCL5 through inhibition of the NF-κB signaling pathway; CCL5 is essential for glioma formation and growth [33]. Decorin interacts with vascular endothelial growth factor receptor 2 (VEGFR2), resulting in increased expression of the tumor suppressor gene *PEG3* and subsequent activation of mitophagy and autophagy [34]. Recent studies have shown that decorin may serve as a favorable marker of the response to anti-VEGF therapy in GBM [35]. Experiments in U87MG, T98G, U251, A172, and U118 glioma cell lines demonstrate that decorin overexpression suppresses cancer cell growth, whereas treatment with exogenous decorin inhibits cell adhesion and migration. However, in vitro data cannot unambiguously confirm the behavior of tumor cells in vivo, as they fail to fully replicate the tumor microenvironment and other complex processes [32].

Data are also available on the role of decorin in various solid tumors, including colorectal and breast cancer [28,36]. Decorin has also been shown to promote metastasis through modification of the extracellular matrix, regulation of signaling pathways such as TGF-β, and EGFR, and modulation of the tumor immune microenvironment [25].

### 2.2. Biglycan

**Figure 2:**
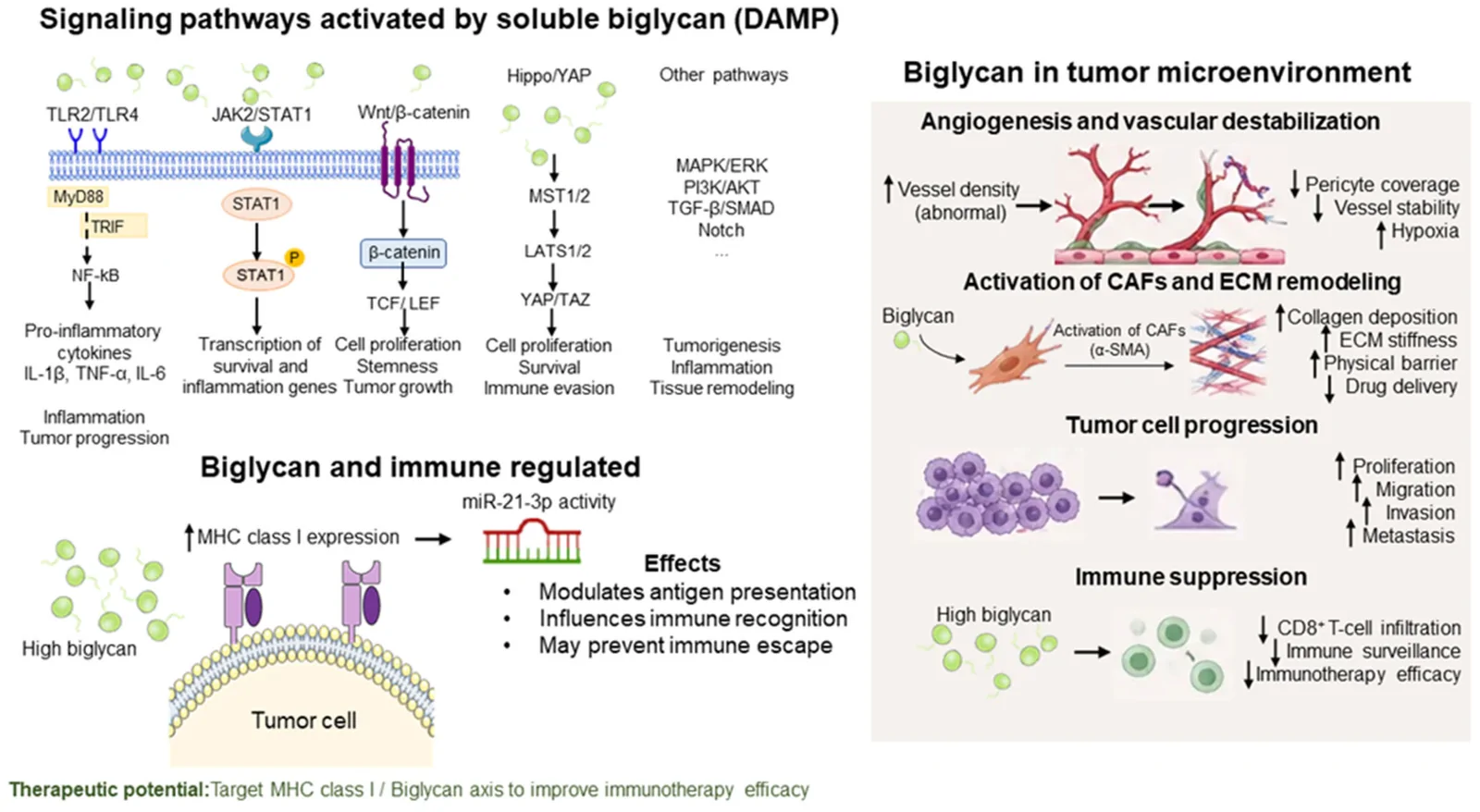
Biglycan as a multifunctional DAMP regulator in carcinogenesis and immune suppression. Stromal biglycan, secreted by tumor endothelial cells, increases matrix stiffness, reduces pericyte coverage of blood vessels, and limits drug access as well as CD8^+^ T-cell infiltration, thereby exacerbating hypoxia and therapy resistance. At the same time, biglycan activates TLR-dependent inflammatory cascades and modulates MHC class I expression, opening up opportunities for targeted intervention on the biglycan/MHC I axis in combination with immunotherapy.

Biglycan is another widely distributed ECM protein and a member of Class I of the small leucine-rich proteoglycan (SLRP) family [37]. It is structurally homologous to decorin [38]. Its 42-kDa core protein, composed of 10 leucine-rich repeats (LRRs), interacts with other ECM proteins, including collagens (types I–IV) and elastin, thereby contributing to the structural stability of the ECM in tissues. Biglycan is released upon tissue injury through the action of proteases. In addition, biglycan can be synthesized de novo by macrophages during inflammation and released into the bloodstream [39,40]. The DAMP signal is initiated by the intact soluble biglycan containing both the protein core and the GAG chain. It is precisely this form, with a mass of ~100–150 kDa, that is capable of binding to TLR2 and TLR4. Acting as a DAMP, biglycan regulates multiple signaling pathways, including TLR, JAK2/STAT1, Wnt/β-catenin, and Hippo/YAP, thereby influencing oncogenesis, inflammation, and tissue remodeling (Figure 2) [41]. Biglycan has been detected in the bloodstream in numerous acute and chronic inflammatory conditions, including lupus nephritis [40], diabetes [42], fibrosis [43], kidney disease [44] and cancer [45].

Biglycan as a component of ECM participates in the regulation of angiogenesis, as well as tumor cell proliferation, migration, and metastasis [46]. Its interaction with toll-like receptors TLR2 and TLR4 triggers a cascade of inflammatory responses and modulates NF-κB activity, processes directly associated with tumor development and progression [47,48].

Stromal biglycan secreted by tumor endothelial cells (TECs) increases tumor blood vessel density and destabilizes vessels by reducing pericyte coverage, thereby impairing tumor blood supply and exacerbating hypoxia [49]. Furthermore, biglycan promotes collagen accumulation and activation of cancer-associated fibroblasts (CAFs), increasing ECM stiffness and hindering drug delivery to the tumor [50]. High levels of biglycan reduce CD8^+^ T-cell infiltration into the tumor, thereby decreasing the efficacy of immune surveillance. Studies using tumor cell lines of various origins have demonstrated that depletion of stromal biglycan normalizes the tumor microenvironment by improving vascular structure, reducing fibrosis, and increasing CD8^+^ T-cell accessibility [48]. This, in turn, enhances the efficacy of standard chemotherapy with paclitaxel. Recent analysis of the Oncomine, Kaplan–Meier Plotter, and cBioPortal databases revealed a correlation between elevated biglycan expression and unfavorable survival in breast cancer (including TNBC), GBM, ependymoma, clear cell renal cell carcinoma, colorectal cancer, gastric cancer, and lung cancer; however, these data represent merely a statistical association and do not prove causality [51]. By contrast, in vitro and in vivo studies have elucidated specific mechanisms: activation of the Wnt/β-catenin pathway via LRP6 in GBM [52], MAPK signaling in ccRCC [53]. Hedgehog–Gli2-dependent regulation in colorectal cancer [54], and exosomal BGN polarizing macrophages towards the M2 phenotype via CXCL10/JAK/STAT1 in gastric cancer [41]. A common molecular link is the capacity of soluble biglycan to act as a DAMP through TLR2/TLR4, thereby creating a pro-inflammatory and immunosuppressive microenvironment, which underscores the need to clearly distinguish between correlative database findings and experimentally verified mechanisms [37]. Biglycan can interact with the LRP6 receptor and activate the Wnt/β-catenin signaling pathway, thereby promoting accelerated tumor cell growth [39]. Conversely, studies of breast cancer metastasis to the brain indicate that biglycan expression is hundreds of times higher in dormant tumor cells and decreases during metastasis. Thus, biglycan is regarded as a potent regulator capable of maintaining cells in a quiescent state and preventing active proliferation and metastasis [55].

Importantly, biglycan can modulate MHC class I expression, thereby influencing the ability of the immune system to recognize and eliminate tumor cells. Elevated biglycan levels correlate with increased MHC class I expression and reduced miR-21-3p activity, indicating a significant protective role of biglycan against tumor immune evasion. This mechanism of immune escape highlights the potential therapeutic value of targeting the MHC class I/biglycan axis to enhance the efficacy of immunotherapy [30].

Biglycan has also been shown to contribute to the activation of pyroptosis in microglia following traumatic spinal cord injury. The interaction of biglycan with TLR4 stimulates the JAK2/STAT1 pathway and increases the expression of the DDX3X protein. DDX3X, in turn, plays a pivotal role in activating the NLRP3 inflammasome and initiating the inflammatory cascade that leads to secondary tissue damage. Furthermore, increased biglycan concentrations have been shown to compensate for deficient TLR4 expression [56,57].

### 2.3. Asporin

Asporin (ASPN) is also known as periodontal ligament-associated protein 1 (PLAP1) [58]. The name asporin derives from the presence of conserved aspartate (D) residues at positions 6–19 in its N-terminal region and its 54% sequence similarity to decorin [59]. To date, ten clinically significant asporin polymorphisms have been identified. Asporin inhibits collagen fibrillogenesis by competing with DCN for collagen binding [60]. The binding of asporin to collagen inhibits collagen mineralization, a process essential for the strength and structural rigidity of various tissues, particularly bone [61]. Its activity as a DAMP is realized through the “naked” core protein (~43 kDa), which binds directly to TLR2 and TLR4 receptors. Extracellular asporin binds directly to the TLR4 on macrophages and dendritic cells. This initiates the MyD88/NF-κB signaling pathway, which leads to the secretion of pro-inflammatory cytokines (TNF-α, IL-6) [62]. ASPN is primarily expressed by cancer-associated fibroblasts or reactive fibroblasts and plays a key role in tumor growth and progression. Stromal fibroblasts have been shown to express ASPN in various cancers, including pancreatic, prostate, gastric, and colorectal cancers [63]. In breast cancer, ASPN plays a dual role, exerting both pro- and antitumor effects through the regulation of TGF-β, EGF, and CD44 signaling pathways [64,65,66]. For example, activation of the TGF-β/Smad2/3 signaling pathway induces the translocation of Smad2/3 into the nucleus, facilitating the transcription of genes that promote epithelial–mesenchymal transition [67,68]. In addition, ASPN activates Rac1 through its interaction with CD44 in fibroblasts and cancer cells, thereby enhancing cell motility and migration [69]. Conversely, in some cases, ASPN inhibits the TGF-β1 receptor, thereby blocking the TGF-β signaling pathway and suppressing tumor proliferation [70]. Similar mechanisms of tumor growth suppression have been demonstrated in low-grade glioma. High expression of this protein is associated with a favorable survival prognosis; however, this effect has not been observed in high-grade glioblastomas [71].

ASPN exerts a broad range of effects in various diseases. For example, it can improve myocardial remodeling in dilated cardiomyopathy and represents a promising therapeutic target in heart failure. It also suppresses collagen matrix-mediated mechanical communication between fibroblasts, thereby improving the prognosis of keloid disease; these effects are also mediated through the TGF-β signaling pathway [72].

### 2.4. Versican

Versican is one of the largest proteoglycans of the extracellular matrix, and in the brain it performs a wide range of functions that change dramatically depending on its isoform, localization, and degree of proteolytic degradation [73]. Versican belongs to the family of leucine-rich proteoglycans (SLRPs) and binds to hyaluronan, forming high-molecular-weight aggregates in the interstitial matrix [74]. Versican is a large chondroitin sulphate proteoglycan (CSPG), a member of the hyalectan family (together with aggrecan, neurocan, and brevican). Its protein core (~400 kDa) contains an N-terminal G1 domain (hyaluronan-binding), a C-terminal G3 domain (with EGF-like repeats and a lectin domain), and a central domain harbouring chondroitin sulphate (CS) chain attachment sites [75]. Intact versican is released from the ECM and recognised by macrophages in a TLR2-dependent manner. It activates the TLR2/TLR6/CD14 complex, initiating the MyD88-dependent pathway and NF-κB, which leads to the production of pro-inflammatory cytokines (TNF-α, IL-6, IL-1β, CCL2) [76]. At the same time, its proteolytic fragment versikine (70 kDa), generated upon cleavage by ADAMTS proteases at the Glu441–Ala442 bond, recruits cDC1 dendritic cells via TLR2 and enhances the anti-tumor CD8+ T-cell response [46,75]. Versican is a key component of perineuronal nets (PNNs) surrounding parvalbumin-positive interneurons. These nets stabilize synapses, restrict plasticity in the adult brain, and protect neurons from oxidative stress [77]. Versican contributes to the maintenance of hydrostatic pressure: owing to its negative charge (heparan sulfate and chondroitin sulfate chains), versican retains water and ions, creating tissue turgor and forming pathways for the diffusion of neurotransmitters and nutrients [78].

Beyond its passive structural function, versican is known to actively engage in interactions with cell surface receptors. It has been demonstrated that versican competes with hyaluronan for binding to CD44, which constitutes the principal receptor for hyaluronan; this competition may, depending on the biological context, result in either the enhancement or the suppression of cellular proliferation and migration, as exemplified in astrocytes. Furthermore, via the arginine-glycine-aspartic acid motif present in its G3 domain, versican is capable of directly binding integrins expressed on neuronal and endothelial membranes, thereby triggering the FAK/ERK signaling cascade and consequently facilitating cell adhesion and survival. In addition, versican has been shown to act as a local reservoir for growth factors such as EGF, FGF-2, VEGF and Bone morphogenetic protein (BMP), establishing concentration gradients that are instrumental in the regulation of neurogenesis within the subventricular zone [79].

Distinct isoforms of versican exert differential functions within the central nervous system. The V0 isoform is abundantly expressed in the embryonic brain, whereas in the adult brain, its presence is largely restricted to sites of inflammation or neoplastic growth. Versican V0 represents the largest isoform and possesses up to 23 glycosaminoglycan (GAG) attachment sites distributed across two attachment regions, designated GAGα and GAGβ [80]. The V1 isoform comprises extended molecules that are predominantly localized to the white matter, including myelinated tracts, as well as in the perastrocytic space. In contrast, the V2 isoform is characterized by relatively short molecules, bearing predominantly chondroitin sulfate chains, and serves as a principal structural component of perineuronal nets in the cerebral cortex and hippocampus. The V3 isoform undergoes minimal glycosylation and is found primarily in basement membranes and the perivascular space [81,82,83]. In addition, a fifth isoform, V4, has recently been characterized; however, its presence in the brain has not yet been confirmed [84,85].

Under pathological conditions, the relative abundance of distinct versican isoforms is frequently altered. Traumatic brain injury and ischemia have been shown to increase the V0/V1 isoform ratio within the matrix of reactive astrocytes, thereby promoting glial scar formation [86]. Within the glial scar, versican not only serves as a physical barrier that impedes axonal regeneration but also fosters secondary inflammatory responses through microglial activation. In epilepsy, a loss of the V2 isoform and concomitant disruption of perineuronal nets have been observed. Such disruption of perineuronal nets leads to hyperexcitability of interneurons and a consequent reduction in the seizure threshold. Furthermore, in Alzheimer’s disease, versican has been demonstrated to bind β-amyloid, facilitating its aggregation while simultaneously impairing peptide clearance via the perivascular pathway (Table 1) [87,88].

Within the tumor microenvironment, versican exhibits context-dependent functional properties. In GBM, overexpression of the V0 and V1 isoforms has been observed, particularly in the peritumoral invasion zone [89]. Versican has been shown to bind EGFR and integrin αVβ3, thereby activating the AKT/mTOR signaling pathway and potently enhancing the proliferation of glioma stem cells [90,91]. Furthermore, versican is capable of promoting angiogenesis via its G3 domain, which stimulates endothelial MMP-9 expression and facilitates the release of sequestered VEGF from ECM, ultimately driving aberrant vessel formation and tumor vascularization. In addition, versican has been demonstrated to induce macrophage polarization toward the M2 (pro-tumorigenic) phenotype through activation of TLR2/TLR6, leading to increased secretion of IL-10 and TGF-β, which in turn suppresses cytotoxic T-lymphocyte activity—a well-characterized mechanism of glioma immune evasion [92]. Notably, the biological actions of versican are closely linked to the activity of metalloproteinases. Tumor cells and activated microglia secrete a range of metalloproteinases, including MMP-1, -2, -3, -9, and ADAMTS-1, -4, -5, which cleave the versican core protein into smaller fragments, particularly within the interglobular domain between G1 and G3 [93].

These proteolytic fragments are no longer capable of binding hyaluronan; however, they expose cryptic epitopes that are recognized by TLR-2 and TLR-4 on macrophages and microglia [76]. This receptor-ligand interaction triggers the NF-κB signaling cascade, culminating in the secretion of pro-inflammatory cytokines, including TNF-α, IL-1β, and IL-6. The ensuing chronic neuroinflammation, in turn, creates a permissive microenvironment that facilitates further tumor invasion and confers resistance to therapy—a phenomenon closely associated with the tumor-associated macrophage (TEM) phenotype [94].

**Table 1:** Involvement of extracellular matrix components in brain function under physiological and pathological conditions.

Name	Functions in the CNS	Role in CNS Pathologies	Key Molecular Mechanisms	References
Tenascin C	Facilitation of neuronal migration during embryogenesis; maintenance of stem cell niches; modulation of cell adhesion; influence on synaptic plasticity.	Tenascin C (~30 kDa): Significantly elevated levels in plasma and cerebrospinal fluid (CSF) in glioblastoma. Associated with high-grade malignancy (WHO grades III–IV) and a mesenchymal phenotype. Levels correlate with poorer survival and recurrence following radiotherapy/chemotherapy. Local elevation of tenascin-C exacerbates inflammation in various CNS injuries, including traumatic brain injury, ischemic stroke, and certain neurodegenerative processes. Modulates neuroinflammation and scar formation (gliosis).	Induces NF-κB activation and the production of pro-inflammatory cytokines (IL-6, TNF-α). Stimulates GBM cell migration and invasion through activation of the FAK/Src signaling pathway. Promotes angiogenesis via VEGF and the formation of an immunosuppressive niche through the recruitment of M2 macrophages.	[95,96,97,98,99]
Heparan Sulfate Proteoglycans (HSPGs)	Stabilization of the blood–brain barrier and the neurovascular interface; facilitation of neurotransmitter storage and delivery within neurons in the form of a “smart” gel; organization of the basement membrane of the neuromuscular junction and support of synaptic plasticity in motor neurons, as well as the synaptic functions of photoreceptors and neurons.	HSPGs (~3–10 kDa) in CNS injury or tumor growth, metalloproteinases and heparanases cleave ectodomains or fragment heparan sulfate chains: Reduce blood–brain barrier (BBB) rigidity through basement membrane degradation, thereby facilitating tumor cell invasion. In glioblastoma stem cells, activate Wnt/β-catenin signaling, thereby supporting self-renewal. Promote angiogenesis	Free heparan sulfate chains act as DAMPs via TLR4/2. Activation of the MyD88-dependent TLR4 pathway leads to the secretion of IL-1β, IL-18, and IFN-β, thereby contributing to neuroinflammation and neuronal damage. The release of bound growth factors, particularly VEGF and FGF-2, serves as a potent stimulus for GBM angiogenesis.	[100,101,102,103]
Asporin (ASPN)	Under physiological conditions, asporin does not perform significant functions in the CNS. It is not a typical component of the brain extracellular matrix but is expressed in normal brain endothelial cells or pericytes.	Asporin (~43 kDa): acts as a growth suppressor in IDH-wild-type glioblastoma. Highly expressed in vascular cells of low-grade gliomas.	In the tumor microenvironment, acts as a growth suppressor of wild-type GBM cells by inhibiting their proliferation, thereby functioning as a negative regulator of tumor growth. Asporin interacts with the TGF-β/Smad2/3 pathway and blocks its pro-proliferative and pro-tumorigenic effects.	[62,71]
Decorin	Stabilization of collagen fibers; neuroprotection; regulation of signaling molecules and growth factors; regulation of neural progenitor migration and differentiation through the control of signaling gradients, primarily TGF-β and BMP; influence on axonal growth and synapse formation; limitation of excessive inflammation; modulation of microglial and astrocytic activity.	Decorin (≈100 kDa): Expressed in the glioblastoma microenvironment and constitutes a component of the “angiogenic matrix.” Maintains an immunosuppressive microenvironment. Can suppress tumor growth through binding to TGF-β. Promotes invasion and tumor progression. Stimulates endothelial cell proliferation, thereby promoting neoangiogenesis. Exerts anti-inflammatory and neuroprotective effects in trauma, ischemia, and neurodegenerative diseases.	During tumor growth and necrosis, decorin fragments are released and act as DAMPs via TLR2 and TLR4, promoting the production of pro-inflammatory cytokines (TNF-α, IL-12p70). Acts as an endogenous ligand for EGFR. Exerts a dual effect on migration, inhibiting it under certain conditions while promoting invasion through MAPK/ERK activation under others.	[14,30,32,35,104,105]
Biglycan	Involvement in bone tissue and ECM formation; organization of collagen fibrils; formation of perineuronal nets; regulation of signaling molecules and growth factors; involvement in development and plasticity.	Biglycan (≈120–130 kDA): serves as a marker of tumor aggressiveness and resistance. Detected in the nuclei of neoplastic glioblastoma cells. Enhances the inflammatory response. Induces epithelial–mesenchymal transition (EMT). Plays a critical role in the development of resistance to temozolomide (TMZ) and supports the proliferation of glioblastoma stem cells (GSCs). Disrupts blood–brain barrier (BBB) integrity. Maintains the glioblastoma stem cell pool through Wnt/β-catenin and Notch signaling.	Acts via TLR2/4 and EGFR, as well as through P2X7 purinoreceptors. Correlates with shorter overall and recurrence-free survival and represents one of the most aggressive DAMPs. It triggers inflammation via TLRs; inflammation and cell death increase ATP levels; ATP amplifies inflammation and IL-1β secretion via P2X7 and NLRP3; and IL-1β and activated EGFR promote tumor cell growth and invasion.	[30,106]
Versican	Stabilizer of synaptic contacts (PNNs) Regulator of diffusion Structural component of the white matter	Versican (~70 kDa): component of the glial scar; impedes regeneration; recruit’s macrophages; inhibits axonal outgrowth Induces proliferation; promotes angiogenesis; exerts immunosuppressive effects (V0/V1 isoforms); supports stem cell maintenance	Binding of versican fragments to TLR2/TLR4 on macrophages, dendritic cells, and other myeloid cells triggers signaling cascades and cytokine release (TNF-α, IL-6, IL-1β), driving neuroinflammation; tumor progression amplifies this loop via further versican release.	[76,94,107]
Hyaluronic Acid	Key component of the brain extracellular matrix Synthesized by neurons and astrocytes under physiological conditions Maintenance of tissue homeostasis Regulation of cell behavior: migration, proliferation, differentiation, and inflammatory responses	Hyaluronic Acid (15–40 kDa): promotes malignancy Via CD44, stimulates invasion, proliferation, and therapy resistance Remodels cellular metabolism Enhances inflammatory responses Impairs remyelination	CD44 binding triggers PI3K/AKT, Ras-MAPK/ERK, and Rho/ROCK signaling cascades, leading to activation of transcription factors (e.g., NF-κB) and upregulation of MMP-2/9 expression; additionally modulates EGFR signaling.	[108,109,110]

### 2.5. Heparan Sulfate Proteoglycans (HSPGs)

Heparan sulfate proteoglycans (HSPGs) consist of a specific core protein covalently linked to glycosaminoglycan chains. These extracellular molecules are widely distributed on cell surfaces, where they interact with a broad range of ligands [111]. In particular, they are essential for the activity of angiogenic factors such as vascular endothelial growth factor A (VEGFA) and fibroblast growth factor 2 (FGF2) [112]. Members of this group are classified according to their cellular localization: syndecans (SDCs) and glypicans (GPCs) are membrane-associated, whereas perlecan (HSPG2), agrin (AGRN), and collagen type XVIII (COL18A1) are secreted into the extracellular matrix [113]. HSPGs act as a source of DAMPs through low-molecular-weight HS fragments generated by heparanase. HS fragments are typically oligosaccharides of 10 to ~20 sugar residues with a molecular mass of ~3–10 kDa, which trigger TLR4-dependent production of IL-1β, IL-6, IL-8, and TNF. By contrast, large intact HS chains (12–50 kDa and above) within the composition of HSPGs may not activate TLR4 but instead bind and neutralize DAMPs (e.g., HMGB1 and histones) [9,114].

HSPGs play a central role in cell adhesion, signaling, and neuronal development and participate in processes such as axonal growth and synapse formation [101,115]. The HS backbone is synthesized by glycosyltransferases of the exostosin family, including Ext1, Ext2, and others. In particular, Ext1 is essential for HS synthesis [116,117]. Cells lacking a functional Ext1 allele do not synthesize HS, and Ext1 knockout mice die during early embryogenesis due to defects in gastrulation [118]. Numerous studies have demonstrated significant changes in the composition and content of this class of substances in the brain throughout development and aging.

Studies indicate that HSPGs play a significant role in the pathogenesis of Alzheimer’s disease. HSPGs have been detected in diffuse and neuritic plaques containing amyloid beta (Aβ). Heparan sulfate inhibits the degradation of fibrillar Aβ, thereby promoting the accumulation and stability of amyloid plaques [119,120].

HSPGs are present in neurofibrillary tangles (NFTs) associated with tauopathies. Modifications of heparan sulfate, particularly 3-O-sulfation, enhance Tau phosphorylation, thereby promoting its pathological transformation [121]. One mechanism of Tau metabolism depends on the activity of mitogen-activated protein kinase 1/2 (ERK1/2). The presence of HSPGs correlates with increased pERK1/2 levels, whereas the absence of HSPGs or disruption of their structure markedly reduces the degree of phosphorylation. Ligand binding to syndecans activates ERK1/2 signaling involved in actin-dependent endocytosis [122]. As co-receptors, HSPGs activate receptor signaling upon cytokine binding, such as the binding of IL-6 to IL-6R. Inhibition of HSPGs reduces IL-6 release [123], whereas inhibition of heparinase reduces IL-6 transcription [124]. HSPGs regulate the interaction of IL-6 with its ligand and receptors, facilitating JAK activation and subsequent ERK1/2 signaling [111]. Inhibition of ERK1/2 reduces IL-6 transcription. HSPG-deficient cells exhibit reduced ERK1/2 activation following the addition of Tau. HSPGs play a critical role in regulating the expression of inflammatory genes in glial cells following the addition of exogenous monomeric Tau through activation of ERK1/2 [125].

Heparan sulfate promotes TRAIL-induced tumor cell apoptosis because TRAIL binds with high affinity to HS on the cell surface, leading to ligand oligomerization and signal amplification. Removal of HS from the surface of MDA-MB-453 breast cancer cells and RPMI-8226 myeloma cells blocked TRAIL-mediated apoptosis. However, long oligosaccharide chains often compete for TRAIL more effectively than short chains. The level of HS/HSPG expression may therefore be an important factor contributing to the sensitivity of tumor cells to TRAIL-induced apoptosis [126].

Chemotherapy with dexamethasone has been shown to suppress heparan sulfate (HS) synthesis by reducing the expression of key enzymes involved in HS biosynthesis, namely N-deacetylase/N-sulfotransferase (NDST1 and NDST2) [127].

Inhibition of FGF2 and its receptor signaling suppresses GBM cell proliferation; HSPGs play an important role in this process by promoting tumor invasion and angiogenesis [128]. Knockout of glycosyltransferases responsible for glycosamine synthesis suppresses tumor growth and increases animal survival in an orthotopic glioma model. At the same time, the tumor cells themselves retain the ability to proliferate actively. The reduced tumor growth is attributable to the suppression of angiogenesis [129].

### 2.6. Hyaluronic Acid

Hyaluronic acid (HA) polymers comprise repeating disaccharide units of glucuronic acid and N-acetyl glucosamine joined by alternating (β-1,3 and β-1,4) glycocidic linkages free of a protein core. An integral component of the ECM, HA polymers are traditionally considered as solvating, structural macromolecules largely responsible for supporting tissue integrity due to substantial viscoelastic properties [130]. HA has been shown to affect both α-amino-3-hydroxyl-5-methyl-4-isoxazolepropionate (AMPA) glutamate receptor mobility and paired-pulse modulation in hippocampal cultures. Another study established a mechanistic link between HA and long-term potentiation (LTP) by showing that removing HA using hyaluronidase (Hyase) suppresses L-typevoltage-dependent Ca^2+^ channel (L-VDCC)-mediated currents in hippocampal slices, reduces Ca^2+^ transients in postsynaptic dendrites and spines, and specifically abolishes an L-VDCC-dependent component of LTP. Furthermore, the injection of Hyase before contextual fear conditioning impairs the formation/retention of fear memories [131,132]. HA fragments (15–40 kDa) and not those from intermediate molecular sizes (75–350 kDa) induced a significant upregulation of the expression of major pro-inflammatory cytokines such as IL-1β, MCP-1 and IL-8 [133].

HA plays a significant role in the development of various pathological mechanisms within the central nervous system, most notably in epilepsy, Alzheimer’s disease, ischemia, and dementia. Depletion of HA in the brain results in a reduction in the extracellular space volume and promotes synchronization of neuronal activity. Epileptiform activity is thought to arise through enhanced ephaptic interactions, elevated extracellular potassium concentrations, and alterations in the metabolism of the primary excitatory neurotransmitter, glutamate. Numerous in vivo and in vitro experiments have provided compelling evidence for the effects of HA reduction on epileptiform activity [134,135]. It is well established that traumatic brain injury, ischemic stroke, and other forms of brain damage that initiate epileptogenesis are associated with an early decrease in high-molecular-weight HA levels; importantly, prevention of this decline has been shown to abrogate epileptogenesis [131,134,136].

In Alzheimer’s disease, HA is involved in multiple mechanisms, including the stabilization of perineuronal nets, the attenuation of the toxic effects of Aβ and hyperphosphorylated tau, and the modulation of neuroinflammation through CD44/RHAMM-dependent signaling pathway [137]. Furthermore, HA levels have been shown to be elevated in the cerebrospinal fluid and blood plasma of AD patients, suggesting a potential role for HA as a diagnostic biomarker; notably, the increase in HA concentration was found to be twice as high in males as in females, whereas no gender-specific differences were observed in the control group [138]. Experimental studies have also demonstrated a reduction in brain HA levels in response to β-amyloid administration in animal models [139]. In Parkinson’s disease (PD), HA has been implicated in the regulation of autophagy, the inhibition of α-synuclein propagation, and the remodeling of the ECM to confer protection on dopaminergic neurons [137].

In the context of the immune system, hyaluronic acid exerts opposing effects depending on its molecular size, and this dual functionality represents a critical mechanism for the regulation of inflammation and tissue repair [140]. In healthy brain, undamaged tissues, hyaluronic acid is present in its high-molecular-weight form (HMW-HA), typically around ~10^7^ Da, and possesses the capacity to sequester water in a volume up to 1000-fold greater than its own. As a fundamental constituent of the glycocalyx, HMW-HA functions as a physical barrier that restricts the passive diffusion of macromolecules and cellular elements, and concurrently exerts anti-angiogenic activity. Accumulating evidence demonstrates that HMW-HA suppresses neovascularization by attenuating both endothelial cell proliferation and migratory capacity. At the cell–matrix interface, HMW-HA engages with cognate surface receptors, thereby diminishing immune cell recognition and effectively impeding macrophage-mediated phagocytosis [130,136].

In the setting of tissue injury (e.g., trauma or inflammation), high-molecular-weight HA undergoes enzymatic degradation by proteolytic enzymes, primarily hyaluronidases, giving rise to smaller fragments that can function as DAMPs. These low-molecular-weight HA fragments are capable of initiating a cascade of inflammatory responses. They are recognized by immune receptors, most notably TLR2 and TLR4. Activation of TLRs leads to the production of pro-inflammatory cytokines, including TNF-α and IL-1β, and stimulates dendritic cell maturation while promoting leukocyte recruitment to the site of injury [141]. Importantly, HA fragments of varying lengths exhibit distinct biological properties. A considerable number of enzymes participate in the enzymatic degradation of HA, among which six specific hyaluronidases (HYAL1, HYAL2, HYAL3, HYAL4, HYALP1, and SPAM1) are the most extensively characterized. Notably, HYAL2 has been shown to be of particular significance in glioma development and progression [142,143]. Elevated HYAL2 expression correlated with poor prognosis in glioma patients, whereas knockdown of HYAL2 led to reduced cell viability and proliferation. Further analysis revealed the induction of apoptosis upon HYAL2 downregulation. Alterations in the levels of proteins regulating the cell cycle (CCNB1, CCND1) and apoptosis (Bcl-2, BAX) corroborated the mechanism of HYAL2 action [142,144]. In addition, experimental studies have demonstrated that components of the hyaluronan matrix stimulate the proliferation of glioma stem cells (CD133^+^). A significant increase in HA, HAS3, and CD44 levels was observed in glioma tissue compared with normal brain tissue, and this elevation correlated with an unfavorable prognosis. Inhibition of HAS3 expression or blockade of CD44 effectively suppressed glioma cell proliferation both in vitro and in animal models. The underlying mechanism was found to involve disruption of autophagic flux and cell cycle arrest at the G1 phase [145,146].

### 2.7. Tenascin C (TNC)

Tenascin C (TNC), a matricellular protein, is a hexameric, multimodular glycoprotein that exists in various molecular forms generated through alternative splicing and post-translational modifications. For tenascin-C, both the intact large molecule (~250–400 kDa) and low-molecular-weight FBG-containing fragments (~30 kDa) are capable of initiating a proinflammatory signal by activating TLR4 [147]. TNC expression is upregulated during embryogenesis, in the immature CNS, and under pathological conditions such as neuroinflammation, injury, and tumorigenesis. TNC has been shown to be present in the glioma microenvironment and glioma stem cell niches and to promote neovascularization, proliferation, invasion, and immunomodulation [99]. The mechanism of TNC action during tumor growth is associated with activation of the PI3K/AKT signaling pathway [148]. TNC is highly expressed in most solid tumors, and increased TNC expression is associated with poor prognosis [149,150].

TNC expression is not limited to tumors of neuronal origin; it is widely observed in various tumor types, including breast cancer, colon adenocarcinoma, prostate adenocarcinoma, and lung carcinoma, and is particularly prominent in gliomas [151]. RNA profiling experiments indicate decreased expression of genes involved in DNA repair and cell cycle regulation in T98G GBM cells under the influence of TNC. Given its characteristic expression patterns, TNC serves as a marker for identifying cells with a stem cell phenotype and as a candidate for targeted therapy. Numerous TNC ligands have been developed, including F16, G11, R6N, PL1, PL3, and Ft; the latter is used in combination with neuropilin-1 to improve the penetration specificity of nanoparticles in antiglioma therapy [152,153].

TNC is widely expressed in neural stem cell niches and plays a role in neurogenesis even in adulthood by maintaining dentate gyrus cells in an immature state and participating in migration upon alterations in WNT signaling [95]. Notably, accumulating evidence suggests that neural stem cells in the subventricular zone may serve as a source of gliomas [154,155,156]. Although fibroblasts are the primary source of TNC in the tumor stroma, in gliomas, TNC is expressed directly by malignant cells [157,158]. Its presence in intracellular vesicles also serves as a marker of tumor proliferation in glioblastoma [159].

TNC interacts with extracellular matrix components, cell surface receptors, and growth factors, thereby influencing various signaling pathways. It binds to fibronectin, periostin, proteoglycans, integrins, and growth factors [160]. TNC peptide fragments generated through proteolysis by matrix metalloproteinases and members of the ADAM family may exert functions distinct from those of the parent molecule [161]. The NOTCH signaling pathway is involved in the regulation of TNC expression [162].

The clinical significance of TNC is evident across a broad range of applications. Tenascin-C serves as a potential prognostic indicator for various types of glioma, including GBM, diffuse intrinsic pontine glioma, and ependymoma [99]. Furthermore, radiolabeled antibodies targeting various TNC domains are undergoing clinical trials for the treatment of malignant gliomas. The TNC-targeting aptamer GBI-10 is used to enhance the efficacy of viral vector therapy [163,164]. Phase II trials of the TNC-targeting IMA950 vaccine have yielded promising results in patients with glioma, demonstrating a median overall survival of 21.2 months [99,165]. A pilot study on 46 patients with glioma using an RNA interference strategy with double-stranded RNA targeting TNC (ATN-RNA) demonstrated a positive effect in the majority of cases, improving overall survival, delaying tumor recurrence, and enhancing health-related quality of life [166]. Despite these advances, further large-scale studies are required for the full implementation of these approaches in clinical practice.

## 3. ECM Components in Glioma Progression

**Figure 3:**
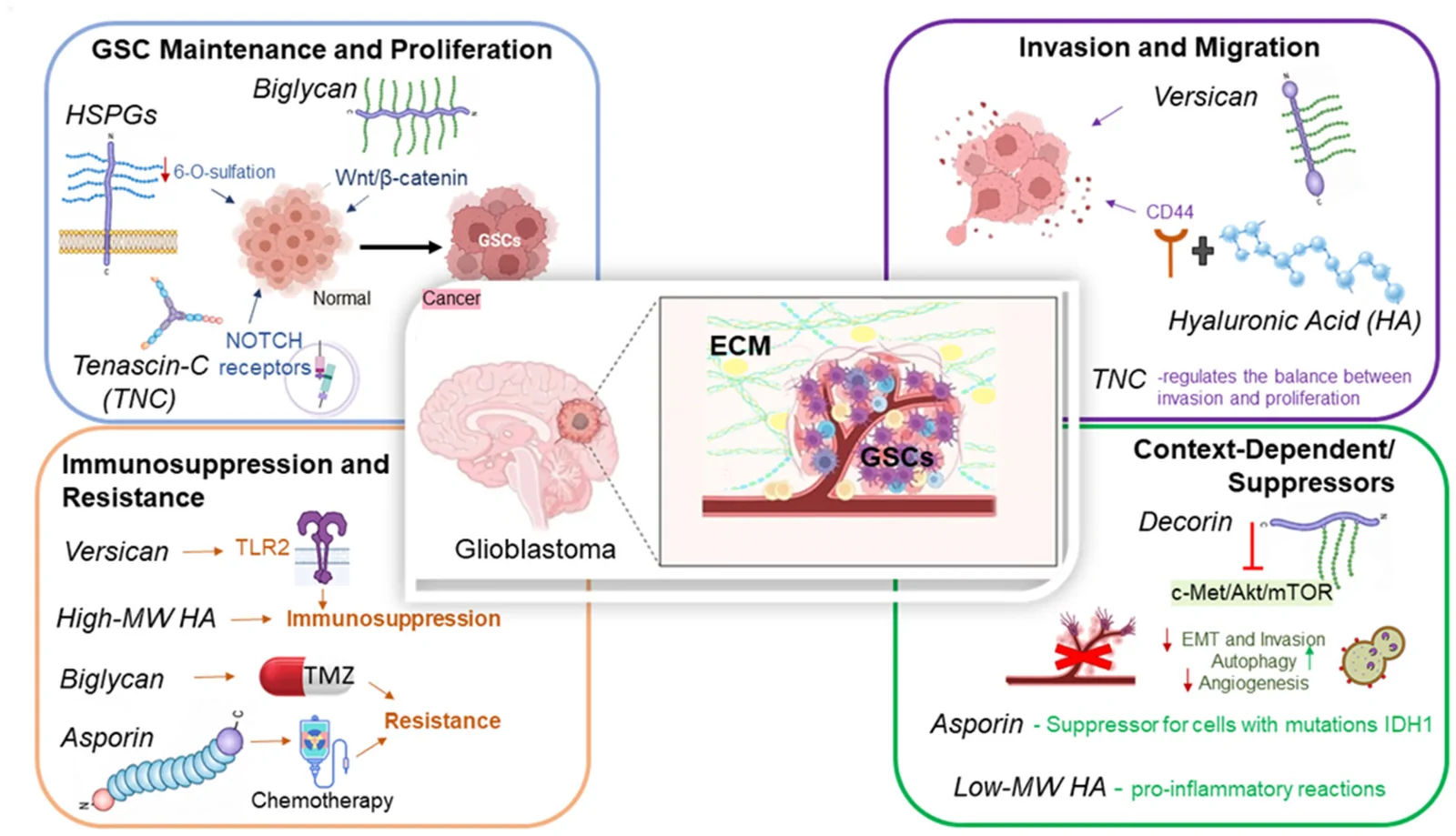
The role of proteoglycans and extracellular matrix glycoproteins in the development of glioblastoma. The schematic diagram depicts the key proteoglycans and glycoproteins of the glioblastoma extracellular matrix—versican, biglycan, decorin, asporin, tenascin-C, hyaluronic acid, and heparan sulfate proteoglycans—which collectively form a complex regulatory network that determines tumor aggressiveness, phenotypic plasticity, and therapeutic resistance. Versican, biglycan, and hyaluronic acid have been shown to activate pro-oncogenic pathways (Wnt, TLR2, CD44) and promote proliferation and stemness, whereas decorin and asporin (under certain contexts) exert tumor-suppressive functions. Tenascin-C and HSPGs, in turn, act as molecular switches between invasion and stemness. Interactions of these ECM components with intracellular signaling cascades (Akt/mTOR, NOTCH, and Wnt/β-catenin) modulate glioma stem cell survival and invasive capacity, thereby opening avenues for combinatorial targeted therapies aimed at overcoming chemotherapy resistance.

ECM plays a crucial role in the functioning and maintenance of brain homeostasis. It is therefore not surprising that it has been incorporated as an integral component of synaptic transmission, as reflected in the concept of the tetrapartite synapse [167,168]. However, particular attention has been drawn to the mechanisms by which ECM components influence tumor progression. Some of these processes are mediated indirectly through effects on immune cells and the tumor microenvironment, whereas others arise from functional alterations of ECM constituents in the presence of malignantly transformed cells [13,160]. In Figure 3, we summarize the available data illustrating the impact of the ECM on the progression of gliomas, which represent the most malignant and prevalent primary brain tumors [169].

Tenascin, biglycan, and decorin can act as potent adjuvants or costimulators by modulating the immune response, whereas others more frequently exhibit immunosuppressive or tolerogenic properties. Why do some DAMPs elicit a response while others do not? The key distinction resides in the “quality” of receptor activation and the downstream signaling cascade. Several levels of regulation can be distinguished.

### 3.1. Divergent Signaling Pathways Downstream of a Single Receptor (TLR4)

TLR4 constitutes the most important mechanism capable of utilizing two principal adaptor proteins: MyD88 and TRIF. The MyD88 pathway initiates rapid and robust activation of NF-κB and MAP kinases, leading to the production of pro-inflammatory cytokines (TNF-α, IL-6, IL-12). This is the classical pathway of immune response activation. The TRIF pathway activates IRF3 and the production of type I interferon (IFN-β), and may also initiate late NF-κB activation. This pathway is frequently associated with a “milder” or even tolerogenic signaling profile [170,171]. Biglycan, upon binding to TLR2/4, predominantly activates the MyD88-dependent pathway, which results in a potent pro-inflammatory response and activation of the NLRP3 inflammasome [37]. Tenascin-C, by contrast, although it activates TLR4, may induce a state of innate immune memory (trained immunity) with elements of tolerance, altering gene expression, which does not invariably lead to full-fledged activation of adaptive immunity [172].

### 3.2. The Role of Co-Receptors and Compartmentalization

The signal emanating from a given receptor may change radically depending on the co-receptor with which it forms a complex. HA, upon binding to TLR4, does not activate it directly. Instead, it forms a TLR4–CD44 complex. This interaction initiates a distinct cascade that leads to the induction of the A20 protein, a potent inhibitor of NF-κB. Consequently, rather than being activated, the TLR4 signal is actively suppressed. This results in HA protecting against LPS-induced septic shock while not itself eliciting inflammation. This is a striking example of how a single receptor can yield opposite outcomes in different contexts [173,174,175].

### 3.3. The Balance of Pro- and Anti-Inflammatory Signals

Many DAMPs can activate several signaling pathways simultaneously. Decorin, upon binding to TLR2/4, activates NF-κB and induces pro-inflammatory cytokines (TNF-α, IL-12). On the other hand, it induces the expression of IL-10—a key anti-inflammatory cytokine. Moreover, decorin is an antagonist of TGF-β, which further modulates the immune response. The net effect depends on the balance of these signals and may shift towards tolerance under certain conditions [31,176,177].

### 3.4. Immunosuppression as an Active Process

Certain molecules do not merely “fail to elicit” a response but actively suppress it. Versican, upon binding to TLR2 on dendritic cells (DCs), initiates a MyD88-dependent but RelB-mediated pathway. This leads to DCs polarization towards an immunosuppressive phenotype that produces IL-10 and IL-6 instead of pro-inflammatory cytokines. Such DCs do not activate an effective T-cell response but, on the contrary, promote immunological tolerance. Furthermore, versican forms a physical barrier within the tumor microenvironment that impedes T-lymphocyte infiltration [178,179,180].

## 4. Discussion

The concept of ICD is currently undergoing active development, accompanied by an expanding understanding of the mechanisms and targets involved in this highly specific process. The classical definition of ICD and the understanding of the role of DAMPs have undergone significant changes. ICD is not merely the release of DAMPs, but a functional process that leads to the activation of the adaptive immune response [181]. ICD is an umbrella term encompassing several forms of regulated cell death modalities, including immunogenic apoptosis, necroptosis, pyroptosis, and ferroptosis [15]. This is precisely why many DAMPs, being important signaling molecules, are not in themselves sufficient inducers of ICD. ICD is a form of regulated cell death that is sufficient to activate the adaptive immune response in immunocompetent syngeneic hosts [182]. The defining criterion is not cell death itself, but the induction of a specific response with the formation of immunological memory. This requires antigenicity (tumor antigens) and adjuvanticity (danger signals). ICD is triggered by specific stressors; other forms of death involving DAMPs do not lead to ICD [183].

To date, more than 40 distinct compounds, including ECM components, have been classified as DAMPs. In this review, we have sought to summarize the available data on the most important components of the brain ECM in the context of neuro-oncology and their roles in ICD. In our view, although all the molecules discussed play significant, and in some cases pivotal, roles in the development and progression of malignant tumors, not all of them qualify as classical DAMPs. Certain ECM molecules promote immunosuppression within the tumor microenvironment and drive oncogenic proliferation.

Current evidence refutes the view of the CNS as an isolated structure and highlights the critical importance of dynamic cell–ECM interactions and the activation of immune mechanisms through DAMPs. In CNS pathologies, including high-grade gliomas and neurodegenerative disorders, ECM components such as tenascin-C, biglycan, decorin, and heparan sulfate proteoglycans versican, and hyaluronic acid shift from serving merely as structural elements to acting as active signaling molecules that modulate the behavior of tumor and glial cells. Under physiological conditions, HSPGs function as co-receptors, regulating growth factor presentation and signaling cascades, including ERK1/2, whereas TNC and small proteoglycans contribute to the maintenance of plasticity, cell migration, and differentiation. Under pathological conditions, the release or structural alteration of these molecules leads to their recognition by innate immune receptors, such as TLR4 and integrins, thereby triggering pro-inflammatory responses through DAMP signaling.

Within the GBM microenvironment, these processes contribute to immunosuppression, enhanced invasion, maintenance of the stem-like cell pool, and therapeutic resistance. In neurodegenerative disorders, DAMP signaling contributes to chronic neuroinflammation, impaired synaptic function, and neuronal death. Post-translational modifications and fragmentation of ECM molecules can further exacerbate these pathological effects.

Therapeutic strategies should therefore be differentiated and focused on the selective modulation of signaling pathways rather than their complete suppression. Blocking pro-tumorigenic TNC fragments, inhibiting DAMP-mediated microglial activation, and regulating HSPG signaling represent promising approaches.

A comprehensive understanding of the kinetics of DAMP release, their conformational changes, and patterns of DAMP–receptor interactions is essential for the development of targeted approaches aimed at restoring immune surveillance, normalizing the signaling environment, and overcoming therapeutic resistance in the CNS. The primary objective is to develop selective modulators capable of finely tuning DAMP signaling without disrupting its physiological functions.

## 5. Conclusions

ICD is not merely the release of molecules, but a strictly programmed process requiring the correct “profile” of DAMPs and the appropriate kinetics of their release. The molecules we have listed (tenascin-C, HSPGs, asporin, decorin, biglycan, versican, and HA) are not in themselves inducers of ICD. They act as modulators of the immune response.

Biglycan and decorin can amplify the immune response by acting as adjuvants. Versican and high-molecular-weight HA actively suppress the immune response, promoting tolerance. Tenascin-C and HSPGs play a more complex, context-dependent role. The distinction between an “inducer” and a “modulator” resides in the capacity to overcome the activation threshold and initiate a full-fledged cascade leading to DC maturation, antigen presentation, and activation of cytotoxic T-lymphocytes. Molecules that are unable to do so either fail to provide a sufficient “danger signal” or actively initiate immunosuppressive programs. The key criterion for a DAMPs molecule is not the induction of ICD but the capacity to activate innate immune receptors (PRRs) [184].

The proteoglycans biglycan and decorin become DAMPs in soluble form following cleavage by MMPs; in the intact state they are bound to collagen and other matrix components. Following cleavage, they become soluble DAMPs and can interact with TLR2 and TLR4 [31,185,186]. Native HMW-HA is immunosuppressive and maintains tolerance. Upon tissue injury, it is cleaved by hyaluronidases into low-molecular-weight fragments (LMW HA), which acquire the capacity to activate TLR2, TLR4, and NLRP3 [187,188,189]. Tenascin-C expression increases under pathological conditions, and it is released from tissues in soluble form, acting as a DAMP via TLR4 [190]. Versican is likewise a DAMP in soluble form (TLR2/6/CD14) [81]. HSPGs, soluble fragments generated upon cleavage by heparinase, become DAMPs via TLR4 [191]. Asporin is poorly studied as a DAMP but likewise exists in soluble form [72]. These molecules are DAMPs because they alert the immune system to tissue damage; however, they do not guarantee the initiation of ICD.

Despite the large number of studies on the pro-oncogenic and anti-oncogenic properties of ECM components, there are to date no drugs introduced into clinical practice that purposefully and selectively modulate the extracellular matrix in the brain. The agents closest to clinical implementation are those aimed at modulating tenascin-C signaling. This renders the field highly promising for the development and creation of novel therapeutic agents.

## Data Availability

No new data were created or analyzed in this study. Data sharing is not applicable to this article.

## Abbreviations

The following abbreviations are used in this manuscript:

Aβ	Amyloid beta
AD	Alzheimer’s disease
ADAMTS	A disintegrin and metalloproteinase with a thrombospondin type 1 motif, member
AGRN	Agrin
AKT	AKT serine/threonine kinase (protein kinase B)
ASPN	Asporin
ATP	Adenosine triphosphate
BBB	Blood–brain barrier
BMP	Bone morphogenetic protein
CAFs	Cancer-associated fibroblasts
CCL5	C-C motif chemokine ligand 5
CD	Cluster of differentiation
CNS	Central nervous system
CSF	Cerebrospinal fluid
DAMPs	Damage-associated molecular patterns
DCN	Decorin
DDX3X	DEAD-box helicases
ECM	Extracellular matrix
EGF	Endothelial growth factor
EGFR	Endothelial growth factor receptor
EMT	Epithelial to mesenchymal transition
ERK	Extracellular signal-regulated kinase
FAK	Focal adhesion kinase
FGF	Fibroblast Growth Factor
GAG	Glycosaminoglycan
GBM	Glioblastoma
GSCs	Glioblastoma stem cells
HA	Hyaluronic acid
HMGB1	High-mobility group box 1
HMW-HA	High-molecular-weight hyaluronic acid
HS	Heparan sulfate
HSPGs	Heparan Sulfate Proteoglycans
HYAL	Hyaluronidases
ICD	Immunogenic cell death
IL-1β	Interleukin-1 beta
JAK2	Janus-kinase 2
LATS1/2	Large tumor suppressor 1/2
LEF	lymphoid enhancer-binding factor
LRP	Low-density lipoprotein receptor-related protein
LRR	Leucine-rich repeat
LTP	Long-term potentiation
L-VDCC	L-type voltage-dependent Ca^2+^ channel
MAPK	Mitogen-activated protein kinase
MEK	Mitogen-activated protein kinase kinase (MAPK/ERK kinase)
MHC I/MHC II	Major histocompatibility complex class I/class II
MMP	Matrix metalloproteinase
MST1/2	Mammalian STE20-like protein kinase 1/2
mTOR	Mechanistic target of rapamycin
NDST1	N-deacetylase
NF-κB	Nuclear factor kappa-light-chain-enhancer of activated B cells
NFTs	Neurofibrillary tangles
NLRP3	NLR family pyrin domain containing 3
NLRs	NOD-like receptors
PD	Parkinson’s disease
PEG3	Paternally expressed gene 3
PNNs	Perineuronal nets
PLAP1	as periodontal ligament-associated protein 1
ROS	Reactive oxygen species
SLRP	Small leucine-rich proteoglycan
STAT1	Signal transducer and activator of transcription 1
TAZ	Transcriptional coactivator with PDZ-binding motif
TCF	T-cell factor
TECs	Tumor endothelial cells
TEM	Tumor-associated macrophage
TGF-β	Thrombocytes grow factor beta
TLRs	Toll-like receptors
TMZ	Temozolomide
TGF-β	Transforming growth factor-beta
TNC	Tenascin C
TNF-α	tumor necrosis factor alpha
TRIF	TIR-domain-containing adaptor-inducing interferon-β
VEGF	Vascular Endothelial Growth Factor
VEGFR2	Vascular endothelial growth factor receptor 2
YAP	Yes-associated protein
α-SMA	α-smooth muscle actin

## References

[1] Jentho E., Weis S. (2021). DAMPs and innate immune training. Front. Immunol..

[2] Lambert C., Zappia J., Sanchez C., Florin A., Dubuc J.-E., Henrotin Y. (2021). The damage-associated molecular patterns (DAMPs) as potential targets to treat osteoarthritis: Perspectives from a review of the literature. Front. Med..

[3] Liu X., Lu F., Chen X. (2022). Examination of the role of necroptotic damage-associated molecular patterns in tissue fibrosis. Front. Immunol..

[4] Lin P.K., Davis G.E. (2023). Extracellular matrix remodeling in vascular disease: Defining its regulators and pathological influence. Arterioscler. Thromb. Vasc. Biol..

[5] Zhao T., Huang Y., Zhu J., Qin Y., Wu H., Yu J., Zhai Q., Li S., Qin X., Wang D. (2025). Extracellular matrix signaling cues: Biological functions, diseases, and therapeutic targets. MedComm.

[6] Bale S., Verma P., Varga J., Bhattacharyya S. (2023). Extracellular matrix–derived damage-associated molecular patterns (DAMP): Implications in systemic sclerosis and fibrosis. J. Investig. Dermatol..

[7] Dzobo K., Dandara C. (2023). The extracellular matrix: Its composition, function, remodeling, and role in tumorigenesis. Biomimetics.

[8] Baghy K., Szakadáti H., Kovalszky I. (2025). Decorin the antifibrotic proteoglycan and its progression in therapy. Am. J. Physiol.-Cell Physiol..

[9] Quan T. (2026). The matrisome as an immunomodulator: A role far beyond its structural support. Biomolecules.

[10] Zhao W., Liu Z., Wu J., Liu A., Yan J. (2025). Potential targets of microglia in the treatment of neurodegenerative diseases: Mechanism and therapeutic implications. Neural Regen. Res..

[11] Rubio C., Pérez-Rubio R., Pérez-Villavicencio J., Serrano-García N., Lee Á., Granados-Rojas L., Tena-Suck M., Rubio-Osornio M. (2026). Dysregulation of the Extracellular Matrix in the Synaptic Pathology of Parkinson’s Disease: Molecular Mechanisms and Novel Therapeutic Approaches. Life.

[12] Simon T., Jackson E., Giamas G. (2020). Breaking through the glioblastoma micro-environment via extracellular vesicles. Oncogene.

[13] Yuan Z., Li Y., Zhang S., Wang X., Dou H., Yu X., Zhang Z., Yang S., Xiao M. (2023). Extracellular matrix remodeling in tumor progression and immune escape: From mechanisms to treatments. Mol. Cancer.

[14] Collado J., Boland L., Ahrendsen J.T., Miska J., Lee-Chang C. (2024). Understanding the glioblastoma tumor microenvironment: Leveraging the extracellular matrix to increase immunotherapy efficacy. Front. Immunol..

[15] Vedunova A.A., Guryev E.L., Gudkov S.V., Mishchenko T.A., Vedunova M.V. (2026). Danger or Salvation? The Role of DAMPs in Cancer Therapy. Cancers.

[16] Ma M., Jiang W., Zhou R. (2024). DAMPs and DAMP-sensing receptors in inflammation and diseases. Immunity.

[17] Lin H., Xiong W., Fu L., Yi J., Yang J. (2025). Damage-associated molecular patterns (DAMPs) in diseases: Implications for therapy. Mol. Biomed..

[18] Pfisterer K., Shaw L.E., Symmank D., Weninger W. (2021). The Extracellular Matrix in Skin Inflammation and Infection. Front Cell Dev. Biol..

[19] Chen S., Birk D.E. (2010). Focus on molecules: Decorin. Exp. Eye Res..

[20] Neill T., Schaefer L., Iozzo R.V. (2012). Decorin: A guardian from the matrix. Am. J. Pathol..

[21] Sun Y., Xu S., Jiang M., Liu X., Yang L., Bai Z., Yang Q. (2021). Role of the extracellular matrix in Alzheimer’s disease. Front. Aging Neurosci..

[22] Jiang R., Smailovic U., Haytural H., Tijms B.M., Li H., Haret R.M., Shevchenko G., Chen G., Abelein A., Gobom J. (2022). Increased CSF-decorin predicts brain pathological changes driven by Alzheimer’s Aβ amyloidosis. Acta Neuropathol. Commun..

[23] Mohan H., Krumbholz M., Sharma R., Eisele S., Junker A., Sixt M., Newcombe J., Wekerle H., Hohlfeld R., Lassmann H. (2010). Extracellular matrix in multiple sclerosis lesions: Fibrillar collagens, biglycan and decorin are upregulated and associated with infiltrating immune cells. Brain Pathol..

[24] Haist V., Ulrich R., Kalkuhl A., Deschl U., Baumgärtner W. (2012). Distinct spatio-temporal extracellular matrix accumulation within demyelinated spinal cord lesions in Theiler’s murine encephalomyelitis. Brain Pathol..

[25] Xie C., Mondal D.K., Ulas M., Neill T., Iozzo R.V. (2022). Oncosuppressive roles of decorin through regulation of multiple receptors and diverse signaling pathways. Am. J. Physiol.-Cell Physiol..

[26] Birch J.L., Coull B.J., Spender L.C., Watt C., Willison A., Syed N., Chalmers A.J., Hossain-Ibrahim M.K., Inman G.J. (2020). Multifaceted transforming growth factor-beta (TGFβ) signalling in glioblastoma. Cell. Signal..

[27] Hu Y., Sun H., Owens R.T., Wu J., Chen Y.Q., Berquin I.M., Perry D., O’Flaherty J.T., Edwards I.J. (2009). Decorin suppresses prostate tumor growth through inhibition of epidermal growth factor and androgen receptor pathways. Neoplasia.

[28] Mao L., Yang J., Yue J., Chen Y., Zhou H., Fan D., Zhang Q., Buraschi S., Iozzo R.V., Bi X. (2021). Decorin deficiency promotes epithelial-mesenchymal transition and colon cancer metastasis. Matrix Biol..

[29] Fuchshofer R., Schneider M., Dillinger A.E., Ohlmann A., Iozzo R.V. (2021). Decorin—An Antagonist of TGF-β in Astrocytes of the Optic Nerve. Int. J. Mol. Sci..

[30] Diehl V., Huber L.S., Trebicka J., Wygrecka M., Iozzo R.V., Schaefer L. (2021). The role of decorin and biglycan signaling in tumorigenesis. Front. Oncol..

[31] Dong Y., Zhong J., Dong L. (2022). The role of decorin in autoimmune and inflammatory diseases. J. Immunol. Res..

[32] Jia Y., Feng Q., Tang B., Luo X., Yang Q., Yang H., Li Q. (2021). Decorin suppresses invasion and EMT phenotype of glioma by Inducing autophagy via c-Met/Akt/mTOR axis. Front. Oncol..

[33] Chatterjee J., Sanapala S., Cobb O., Bewley A., Goldstein A.K., Cordell E., Ge X., Garbow J.R., Holtzman M.J., Gutmann D.H. (2021). Asthma reduces glioma formation by T cell decorin-mediated inhibition of microglia. Nat. Commun..

[34] Neill T., Iozzo R.V. (2022). The role of decorin proteoglycan in mitophagy. Cancers.

[35] Patel K.S., Yao J., Raymond C., Yong W., Everson R., Liau L.M., Nathanson D., Kornblum H., Wang C., Oughourlian T. (2020). Decorin expression is associated with predictive diffusion MR phenotypes of anti-VEGF efficacy in glioblastoma. Sci. Rep..

[36] Hu X., Villodre E.S., Larson R., Rahal O.M., Wang X., Gong Y., Song J., Krishnamurthy S., Ueno N.T., Tripathy D. (2021). Decorin-mediated suppression of tumorigenesis, invasion, and metastasis in inflammatory breast cancer. Commun. Biol..

[37] Appunni S., Rubens M., Ramamoorthy V., Anand V., Khandelwal M., Sharma A. (2021). Biglycan: An emerging small leucine-rich proteoglycan (SLRP) marker and its clinicopathological significance. Mol. Cell. Biochem..

[38] McCoy M.S., Diehl V., Trebicka J., Wygrecka M., Schaefer L. (2021). Biglycan: A regulator of hepatorenal inflammation and autophagy. Matrix Biol..

[39] Berdiaki A., Giatagana E.-M., Tzanakakis G., Nikitovic D. (2023). The landscape of small leucine-rich proteoglycan impact on cancer pathogenesis with a focus on biglycan and lumican. Cancers.

[40] Kunnathattil M., Rahul P., Skaria T. (2024). Soluble vascular endothelial glycocalyx proteoglycans as potential therapeutic targets in inflammatory diseases. Immunol. Cell Biol..

[41] Li W., Wei H., Liu J., Zhao Z., Wang F., Qiao L., Yin S., Zhang C., Huo M. (2025). Exosomal Biglycan promotes gastric cancer progression via M2 polarization and CXCL10-mediated JAK/STAT1 activation. Cancer Lett..

[42] Yusop N., Moseley R., Waddington R.J. (2023). Hyperglycemia exerts disruptive effects on the secretion of TGF-β1 and its matrix ligands, decorin and biglycan, by mesenchymal sub-populations and macrophages during bone repair. Front. Dent. Med..

[43] Scuruchi M., Mannino F., Imbesi C., Pallio G., Vermiglio G., Bagnato G., Minutoli L., Bitto A., Squadrito F., Irrera N. (2023). Biglycan involvement in heart fibrosis: Modulation of adenosine 2A receptor improves damage in immortalized cardiac fibroblasts. Int. J. Mol. Sci..

[44] Wei A., Zhang Y., Xu Y., Schneider J., Detterman N., Shu X., Gregersen T., Folch M.N., Li Y., Chen S. (2026). TLR2 Activation Enhances Inflammatory Signaling and Accelerates Cyst Growth and Renal Fibrosis in Polycystic Kidney Disease Mice. Int. J. Mol. Sci..

[45] Zheng S., Liang J.Y., Tang Y., Xie J., Zou Y., Yang A., Shao N., Kuang X., Ji F., Liu X. (2023). Dissecting the role of cancer-associated fibroblast-derived biglycan as a potential therapeutic target in immunotherapy resistance: A tumor bulk and single-cell transcriptomic study. Clin. Transl. Med..

[46] Deb G., Cicala A., Papadas A., Asimakopoulos F. (2022). Matrix proteoglycans in tumor inflammation and immunity. Am. J. Physiol.-Cell Physiol..

[47] Cong L., Maishi N., Annan D.A., Young M.F., Morimoto H., Morimoto M., Nam J.-M., Hida Y., Hida K. (2021). Inhibition of stromal biglycan promotes normalization of the tumor microenvironment and enhances chemotherapeutic efficacy. Breast Cancer Res..

[48] Yu L., Maishi N., Matsuda A., Hida K. (2023). The functional network of biglycan: A new frontier in tumor progression. Proteoglycan Res..

[49] Thomas J.A., Gireesh Moly A.G., Xavier H., Suboj P., Ladha A., Gupta G., Singh S.K., Palit P., Babykutty S. (2023). Enhancement of immune surveillance in breast cancer by targeting hypoxic tumor endothelium: Can it be an immunological switch point?. Front. Oncol..

[50] Buruiană A., Gheban B.-A., Gheban-Roșca I.-A., Georgiu C., Crișan D., Crișan M. (2024). The tumor stroma of squamous cell carcinoma: A complex environment that fuels cancer progression. Cancers.

[51] Zhao S.-F., Yin X.-J., Zhao W.-J., Liu L.-C., Wang Z.-P. (2020). Biglycan as a potential diagnostic and prognostic biomarker in multiple human cancers. Oncol. Lett..

[52] Mirzaei R., D’Mello C., Liu M., Nikolic A., Kumar M., Visser F., Bose P., Gallo M., Yong V.W. (2023). Single-Cell Spatial Analysis Identifies Regulators of Brain Tumor–Initiating Cells. Cancer Res..

[53] Xia H., He T., Li X., Zhao K., Zhang Z., Zhu G., Yang H., Yan X., Wang Q., Li Z. (2025). Study on the mechanism of BGN in progression and metastasis of ccRCC. BMC Med. Genom..

[54] Zeng S., Zhou F., Wang Y., Zhai Z., Xu L., Wang H., Chen X., Luo S., Cheng M. (2021). Aberrant expression of the extracellular matrix component Biglycan regulated by Hedgehog signalling promotes colorectal cancer cell proliferation: Hedgehog signalling regulates the expression of BGN. Acta Biochim. Biophys. Sin..

[55] Manupati K., Paul R., Hao M., Haas M., Bian Z.C., Holm T.M., Guan J.-L., Yeo S.K. (2022). Biglycan promotes cancer stem cell properties, NFκB signaling and metastatic potential in breast cancer cells. Cancers.

[56] Wang J., Zhang F., Xu H., Yang H., Shao M., Xu S., Lyu F. (2022). TLR4 aggravates microglial pyroptosis by promoting DDX3X-mediated NLRP3 inflammasome activation via JAK2/STAT1 pathway after spinal cord injury. Clin. Transl. Med..

[57] Huang M., Yao G., He B., Zhou X., Liu G., Dong W. (2025). Microglia and programmed cell death in spinal cord injury: Beyond apoptosis. Front. Cell Dev. Biol..

[58] Das N. (2025). Periodontal Ligament-associated Protein-1/Asporin in Periodontal Health: A Crucial Regulator of Oral and Systemic Function. Indian J. Dent. Sci..

[59] Dhungyal B., Koirala P. (2025). Regulatory Role and Significance of Class I SLRPs: Biglycan, Decorin, and Asporin. The Biological Role of Small Leucine-Rich Proteoglycans (SLRPs) in Matrix Homeostasis and Diseases.

[60] Duval E., Bigot N., Hervieu M., Kou I., Leclercq S., Galéra P., Boumediene K., Baugé C. (2011). Asporin expression is highly regulated in human chondrocytes. Mol. Med..

[61] Ikegawa S. (2008). Expression, regulation and function of asporin, a susceptibility gene in common bone and joint diseases. Curr. Med. Chem..

[62] Zhan S., Li J., Ge W. (2019). Multifaceted roles of asporin in cancer: Current understanding. Front. Oncol..

[63] Itoh G., Takagane K., Fukushi Y., Kuriyama S., Umakoshi M., Goto A., Yanagihara K., Yashiro M., Tanaka M. (2022). Cancer-associated fibroblasts educate normal fibroblasts to facilitate cancer cell spreading and T-cell suppression. Mol. Oncol..

[64] Maris P., Blomme A., Palacios A.P., Costanza B., Bellahcène A., Bianchi E., Gofflot S., Drion P., Trombino G.E., Di Valentin E. (2015). Asporin is a fibroblast-derived TGF-β1 inhibitor and a tumor suppressor associated with good prognosis in breast cancer. PLoS Med..

[65] Tanaka M. (2022). Crosstalk of tumor stromal cells orchestrates invasion and spreading of gastric cancer. Pathol. Int..

[66] Karagiorgou Z., Fountas P.N., Manou D., Knutsen E., Theocharis A.D. (2022). Proteoglycans determine the dynamic landscape of EMT and cancer cell stemness. Cancers.

[67] Moustakas A., Heldin C.-H. (2016). Mechanisms of TGFβ-induced epithelial–mesenchymal transition. J. Clin. Med..

[68] Chen F., Lyu L., Xing C., Chen Y., Hu S., Wang M., Ai Z. (2025). The pivotal role of TGF-β/Smad pathway in fibrosis pathogenesis and treatment. Front. Oncol..

[69] Sasaki Y., Takagane K., Konno T., Itoh G., Kuriyama S., Yanagihara K., Yashiro M., Yamada S., Murakami S., Tanaka M. (2021). Expression of asporin reprograms cancer cells to acquire resistance to oxidative stress. Cancer Sci..

[70] Hernandez K., Nguyen C.H., Rijal G. (2025). Asporin increases the extracellular matrix cross-links and inhibits the cancer cell migration. Tumor Biol..

[71] Muthukrishnan S.D., Qi H., Wang D., Elahi L., Pham A., Alvarado A.G., Li T., Gao F., Kawaguchi R., Lai A. (2024). Low-and high-grade glioma-associated vascular cells differentially regulate tumor growth. Mol. Cancer Res..

[72] Fan R., Yan X., Zhang W. (2022). Relationship between asporin and extracellular matrix behavior: A literature review. Medicine.

[73] Islam S., Watanabe H. (2020). Versican: A dynamic regulator of the extracellular matrix. J. Histochem. Cytochem..

[74] Bignami A., Hosley M., Dahl D. (1993). Hyaluronic acid and hyaluronic acid-binding proteins in brain extracellular matrix. Anat. Embryol..

[75] Hatano S., Watanabe H. (2020). Regulation of macrophage and dendritic cell function by chondroitin sulfate in innate to antigen-specific adaptive immunity. Front. Immunol..

[76] Garantziotis S., Savani R.C. (2022). Proteoglycans in Toll-like receptor responses and innate immunity. Am. J. Physiol.-Cell Physiol..

[77] Fawcett J.W., Oohashi T., Pizzorusso T. (2019). The roles of perineuronal nets and the perinodal extracellular matrix in neuronal function. Nat. Rev. Neurosci..

[78] Silva P.L., Negrini D., Rocco P.R.M. (2015). Mechanisms of ventilator-induced lung injury in healthy lungs. Best Pract. Res. Clin. Anaesthesiol..

[79] Tang F., Brune J.E., Chang M.Y., Reeves S.R., Altemeier W.A., Frevert C.W. (2022). Defining the versican interactome in lung health and disease. Am. J. Physiol.-Cell Physiol..

[80] Yamagata M., Sanes J.R. (2005). Versican in the developing brain: Lamina-specific expression in interneuronal subsets and role in presynaptic maturation. J. Neurosci..

[81] Wight T.N., Kang I., Evanko S.P., Harten I.A., Chang M.Y., Pearce O.M.T., Allen C.E., Frevert C.W. (2020). Versican—A Critical Extracellular Matrix Regulator of Immunity and Inflammation. Front. Immunol..

[82] Tewari B.P., Chaunsali L., Prim C.E., Sontheimer H. (2022). A glial perspective on the extracellular matrix and perineuronal net remodeling in the central nervous system. Front. Cell. Neurosci..

[83] Schmalfeldt M., Dours-Zimmermann M.a.T., Winterhalter K.H., Zimmermann D.R. (1998). Versican V2 is a major extracellular matrix component of the mature bovine brain. J. Biol. Chem..

[84] Kischel P., Waltregny D., Dumont B., Turtoi A., Greffe Y., Kirsch S., De Pauw E., Castronovo V. (2010). Versican overexpression in human breast cancer lesions: Known and new isoforms for stromal tumor targeting. Int. J. Cancer.

[85] Mohamedi Y., Fontanil T., Cobo T., Cal S., Obaya A.J. (2020). New insights into ADAMTS metalloproteases in the central nervous system. Biomolecules.

[86] Kwok J.C.F., Dityatev A. (2026). Extracellular matrix remodelling in neurological diseases. Nat. Rev. Neurol..

[87] Pintér P., Alpár A. (2022). The role of extracellular matrix in human neurodegenerative diseases. Int. J. Mol. Sci..

[88] Amontree M., Deasy S., Turner R.S., Conant K. (2023). Matrix disequilibrium in Alzheimer’s disease and conditions that increase Alzheimer’s disease risk. Front. Neurosci..

[89] Jayaram M.A., Phillips J.J. (2024). Role of the microenvironment in glioma pathogenesis. Annu. Rev. Pathol. Mech. Dis..

[90] Popova N.V., Jücker M. (2022). The functional role of extracellular matrix proteins in cancer. Cancers.

[91] Liu X., Liu Y., Qi Y., Huang Y., Hu F., Dong F., Shu K., Lei T. (2022). Signal pathways involved in the interaction between tumor-associated macrophages/TAMs and glioblastoma cells. Front. Oncol..

[92] Xu Y., Benedikt J., Ye L. (2024). Hyaluronic acid interacting molecules mediated crosstalk between cancer cells and microenvironment from primary tumour to distant metastasis. Cancers.

[93] Nilović V. (2023). Brain Extracellular Matrix and the Connection with Neuropsychiatric Disorders.

[94] Wight T.N., Kang I., Merrilees M.J. (2014). Versican and the control of inflammation. Matrix Biol..

[95] Tucić M., Stamenković V., Andjus P. (2021). The extracellular matrix glycoprotein tenascin C and adult neurogenesis. Front. Cell Dev. Biol..

[96] Salviano-Silva A., Wollmann K., Brenna S., Reimer R., Neumann J.E., Dottermusch M., Woythe L., Maire C.L., Puig B., Schüller U. (2025). Extracellular Vesicles Carrying Tenascin-C are Clinical Biomarkers and Improve Tumor-Derived DNA Analysis in Glioblastoma Patients. ACS Nano.

[97] Manrique-Castano D., Dzyubenko E., Borbor M., Vasileiadou P., Kleinschnitz C., Roll L., Faissner A., Hermann D.M. (2021). Tenascin-C preserves microglia surveillance and restricts leukocyte and, more specifically, T cell infiltration of the ischemic brain. Brain Behav. Immun..

[98] Jin Y.-L., Bao S.-W., Huang M.-X., Gao Y.-J., Lu H.-J., Wu X.-B. (2025). The role of tenascin-C in neuroinflammation and neuroplasticity. Int. J. Mol. Sci..

[99] Fu Z., Zhu G., Luo C., Chen Z., Dou Z., Chen Y., Zhong C., Su S., Liu F. (2022). Matricellular protein tenascin C: Implications in glioma progression, gliomagenesis, and treatment. Front. Oncol..

[100] Melrose J. (2024). CNS/PNS proteoglycans functionalize neuronal and astrocyte niche microenvironments optimizing cellular activity by preserving membrane polarization dynamics, ionic microenvironments, ion fluxes, neuronal activation, and network neurotransductive capacity. J. Neurosci. Res..

[101] Melrose J. (2024). Hippo cell signaling and HS-proteoglycans regulate tissue form and function, age-dependent maturation, extracellular matrix remodeling, and repair. Am. J. Physiol. Cell Physiol..

[102] Barré L., Shaukat I., Ouzzine M. (2025). Fam20B Gain-of-Function Blocks The synthesis of Glycosaminoglycan Chains of Proteoglycans and inhibits proliferation and migration of glioblastoma cells. Cells.

[103] Fawcett J.W., Kwok J.C. (2022). Proteoglycan sulphation in the function of the mature central nervous system. Front. Integr. Neurosci..

[104] Siddiqui N., Oshima K., Hippensteel J.A. (2022). Proteoglycans and glycosaminoglycans in central nervous system injury. Am. J. Physiol.-Cell Physiol..

[105] Ma H.-I., Hueng D.-Y., Shui H.-A., Han J.-M., Wang C.-H., Lai Y.-H., Cheng S.-Y., Xiao X., Chen M.-T., Yang Y.-P. (2014). Intratumoral decorin gene delivery by AAV vector inhibits brain glioblastomas and prolongs survival of animals by inducing cell differentiation. Int. J. Mol. Sci..

[106] Okonechnikov K., Ghasemi D.R., Schrimpf D., Tonn S., Mynarek M., Koster J., Milde T., Zheng T., Sievers P., Sahm F. (2025). Biglycan-driven risk stratification in ZFTA-RELA fusion supratentorial ependymomas through transcriptome profiling. Acta Neuropathol. Commun..

[107] Vigo-Díaz N., López-Cortés R., Velo-Heleno I., Rodríguez-Silva L., Núñez C. (2025). Proteoglycans in breast cancer: Friends and foes. Biomolecules.

[108] Safarians G., Sohrabi A., Solomon I., Xiao W., Bastola S., Rajput B.W., Epperson M., Rosenzweig I., Tamura K., Singer B. (2023). Glioblastoma Spheroid Invasion through Soft, Brain-Like Matrices Depends on Hyaluronic Acid–CD44 Interactions. Adv. Healthc. Mater..

[109] Catania G., Rodella G., Vanvarenberg K., Préat V., Malfanti A. (2023). Combination of hyaluronic acid conjugates with immunogenic cell death inducer and CpG for glioblastoma local chemo-immunotherapy elicits an immune response and induces long-term survival. Biomaterials.

[110] Yan T., Wang K., Li J., Hu H., Yang H., Cai M., Liu R., Li H., Wang N., Shi Y. (2022). Suppression of the hyaluronic acid pathway induces M1 macrophages polarization via STAT1 in glioblastoma. Cell Death Discov..

[111] Matsuzaka Y., Yashiro R. (2024). Classification and molecular functions of heparan sulfate proteoglycans and their molecular mechanisms with the receptor. Biologics.

[112] Greco N., Masola V., Onisto M. (2025). Heparan sulfate proteoglycans (HSPGs) and their degradation in health and disease. Biomolecules.

[113] Farrugia B.L., Melrose J. (2023). The glycosaminoglycan side chains and modular core proteins of heparan sulphate proteoglycans and the varied ways they provide tissue protection by regulating physiological processes and cellular behaviour. Int. J. Mol. Sci..

[114] Goodall K.J., Poon I.K., Phipps S., Hulett M.D. (2014). Soluble heparan sulfate fragments generated by heparanase trigger the release of pro-inflammatory cytokines through TLR-4. PLoS ONE.

[115] Hayes A.J., Melrose J. (2023). HS, an ancient molecular recognition and information storage glycosaminoglycan, equips HS-proteoglycans with diverse matrix and cell-interactive properties operative in tissue development and tissue function in health and disease. Int. J. Mol. Sci..

[116] Annaval T., Wild R., Crétinon Y., Sadir R., Vivès R.R., Lortat-Jacob H. (2020). Heparan sulfate proteoglycans biosynthesis and post synthesis mechanisms combine few enzymes and few core proteins to generate extensive structural and functional diversity. Molecules.

[117] Leisico F., Omeiri J., Le Narvor C., Beaudouin J., Hons M., Fenel D., Schoehn G., Couté Y., Bonnaffé D., Sadir R. (2022). Structure of the human heparan sulfate polymerase complex EXT1-EXT2. Nat. Commun..

[118] Kinoshita T., Tomita H., Okada H., Niwa A., Hyodo F., Kanayama T., Matsuo M., Imaizumi Y., Kuroda T., Hatano Y. (2021). Endothelial cell-specific reduction of heparan sulfate suppresses glioma growth in mice. Discov. Oncol..

[119] Snow A.D., Cummings J.A., Lake T. (2021). The unifying hypothesis of Alzheimer’s disease: Heparan sulfate proteoglycans/glycosaminoglycans are key as first hypothesized over 30 years ago. Front. Aging Neurosci..

[120] Ozsan McMillan I., Li J.-P., Wang L. (2023). Heparan sulfate proteoglycan in Alzheimer’s disease: Aberrant expression and functions in molecular pathways related to amyloid-β metabolism. Am. J. Physiol.-Cell Physiol..

[121] Mah D., Zhao J., Liu X., Zhang F., Liu J., Wang L., Linhardt R., Wang C. (2021). The sulfation code of tauopathies: Heparan sulfate proteoglycans in the prion like spread of tau pathology. Front. Mol. Biosci..

[122] Lake D., Corrêa S.A., Müller J. (2016). Negative feedback regulation of the ERK1/2 MAPK pathway. Cell. Mol. Life Sci..

[123] Alhendi A., Naser S.A. (2023). The dual role of interleukin-6 in Crohn’s disease pathophysiology. Front. Immunol..

[124] Coombe D.R., Gandhi N.S. (2019). Heparanase: A challenging cancer drug target. Front. Oncol..

[125] Song L., Oseid D.E., Wells E.A., Coaston T., Robinson A.S. (2022). Heparan sulfate proteoglycans (HSPGs) serve as the mediator between monomeric tau and its subsequent intracellular ERK1/2 pathway activation. J. Mol. Neurosci..

[126] Luo Y., Hao H., Wang Z., Ong C.Y., Dutcher R., Xu Y., Liu J., Pedersen L.C., Xu D. (2024). Heparan sulfate promotes TRAIL-induced tumor cell apoptosis. Elife.

[127] Veraldi N., Zouggari N., de Agostini A. (2020). The challenge of modulating heparan sulfate turnover by multitarget heparin derivatives. Molecules.

[128] Ferguson H.R., Smith M.P., Francavilla C. (2021). Fibroblast growth factor receptors (FGFRs) and noncanonical partners in cancer signaling. Cells.

[129] Peixoto A., Relvas-Santos M., Azevedo R., Santos L.L., Ferreira J.A. (2019). Protein Glycosylation and Tumor Microenvironment Alterations Driving Cancer Hallmarks. Front. Oncol..

[130] Petrey A.C., de la Motte C.A. (2014). Hyaluronan, a crucial regulator of inflammation. Front. Immunol..

[131] Muto J., Sayama K., Gallo R.L., Kimata K. (2019). Emerging evidence for the essential role of hyaluronan in cutaneous biology. J. Dermatol. Sci..

[132] Vedunova M., Sakharnova T., Mitroshina E., Perminova M., Zakharov Y., Pimashkin A., Dityatev A., Mukhina I. (2013). Seizure-like activity in hyaluronidase-treated dissociated hippocampal cultures. Front. Cell. Neurosci..

[133] Romo M., López-Vicario C., Pérez-Romero N., Casulleras M., Martínez-Puchol A.I., Sánchez B., Flores-Costa R., Alcaraz-Quiles J., Duran-Güell M., Ibarzabal A. (2022). Small fragments of hyaluronan are increased in individuals with obesity and contribute to low-grade inflammation through TLR-mediated activation of innate immune cells: Molecular Biology. Int. J. Obes..

[134] Perkins K.L., Arranz A.M., Yamaguchi Y., Hrabetova S. (2017). Brain extracellular space, hyaluronan, and the prevention of epileptic seizures. Rev. Neurosci..

[135] Balashova A., Pershin V., Zaborskaya O., Tkachenko N., Mironov A., Guryev E., Kurbatov L., Gainullin M., Mukhina I. (2019). Enzymatic Digestion of Hyaluronan-Based Brain Extracellular Matrix in vivo Can Induce Seizures in Neonatal Mice. Front. Neurosci..

[136] Audam T.N., Howard C.M., Little D.T., Garrett L.F., Zheng Y.W., Gu Z., Brittian K.R., Gray R., Chariker J., Singhal R.A. (2025). Hyaluronan provokes inflammation but suppresses phagocytotic function in macrophages. J. Mol. Cell. Cardiol..

[137] Wang Y., Duan J., Zang L., Sun T., Lv H., Liu F., Ma X. (2026). Hyaluronic acid: Emerging roles and biomaterial innovations in Alzheimer’s and Parkinson’s disease therapy. Front. Pharmacol..

[138] Nielsen H.M., Palmqvist S., Minthon L., Londos E., Wennstrom M. (2012). Gender-dependent levels of hyaluronic acid in cerebrospinal fluid of patients with neurodegenerative dementia. Curr. Alzheimer Res..

[139] Genedani S., Agnati L.F., Leo G., Buzzega D., Maccari F., Carone C., Andreoli N., Filaferro M., Volpi N. (2010). β-Amyloid Fibrillation and/or Hyperhomocysteinemia Modify Striatal Patterns of Hyaluronic Acid and Dermatan Sulfate: Possible Role in the Pathogenesis of Alzheimer’s Disease. Curr. Alzheimer Res..

[140] Litwiniuk M., Krejner A., Speyrer M.S., Gauto A.R., Grzela T. (2016). Hyaluronic acid in inflammation and tissue regeneration. Wounds.

[141] Ferreira N.d.R., Sanz C.K., Raybolt A., Pereira C.M., DosSantos M.F. (2022). Action of hyaluronic acid as a damage-associated molecular pattern molecule and its function on the treatment of temporomandibular disorders. Front. Pain Res..

[142] Yan T., Yang H., Xu C., Liu J., Meng Y., Jiang Q., Li J., Kang G., Zhou L., Xiao S. (2023). Inhibition of hyaluronic acid degradation pathway suppresses glioma progression by inducing apoptosis and cell cycle arrest. Cancer Cell Int..

[143] Hamester F., Stürken C., Legler K., Eylmann K., Möller K., Roßberg M., Gorzelanny C., Bauer A.T., Windhorst S., Schmalfeldt B. (2022). Key Role of Hyaluronan Metabolism for the Development of Brain Metastases in Triple-Negative Breast Cancer. Cells.

[144] Gousias K., Theocharous T., Simon M. (2022). Mechanisms of Cell Cycle Arrest and Apoptosis in Glioblastoma. Biomedicines.

[145] Chen J.-W.E., Leary S., Barnhouse V., Sarkaria J.N., Harley B.A. (2022). Matrix hyaluronic acid and hypoxia influence a CD133+ subset of patient-derived glioblastoma cells. Tissue Eng. Part A.

[146] Ruiz-Garcia H., Alvarado-Estrada K., Schiapparelli P., Quinones-Hinojosa A., Trifiletti D.M. (2020). Engineering Three-Dimensional Tumor Models to Study Glioma Cancer Stem Cells and Tumor Microenvironment. Front. Cell. Neurosci..

[147] Gaule T., Simmons K.J., Walker K., Del Galdo F., Ross R.L., Viswambharan H., Krishnappa J., Pacey J., McPhillie M., Tomlinson D.C. (2025). Targeting Tenascin-C-Toll-like Receptor 4 signalling with Adhiron-derived small molecules-a viable strategy for reducing fibrosis in Systemic Sclerosis. Bioorganic Chem..

[148] Zhang Q., Xu B., Hu F., Chen X., Liu X., Zhang Q., Zuo Y. (2021). Tenascin C promotes glioma cell malignant behavior and inhibits chemosensitivity to paclitaxel via activation of the PI3K/AKT signaling pathway. J. Mol. Neurosci..

[149] Li Z.-L., Zhang H.-L., Huang Y., Huang J.-H., Sun P., Zhou N.-N., Chen Y.-H., Mai J., Wang Y., Yu Y. (2020). Autophagy deficiency promotes triple-negative breast cancer resistance to T cell-mediated cytotoxicity by blocking tenascin-C degradation. Nat. Commun..

[150] Wawrzyniak D., Grabowska M., Głodowicz P., Kuczyński K., Kuczyńska B., Fedoruk-Wyszomirska A., Rolle K. (2020). Down-regulation of tenascin-C inhibits breast cancer cells development by cell growth, migration, and adhesion impairment. PLoS ONE.

[151] Liot S., Aubert A., Hervieu V., El Kholti N., Schalkwijk J., Verrier B., Valcourt U., Lambert E. (2020). Loss of Tenascin-X expression during tumor progression: A new pan-cancer marker. Matrix Biol. Plus.

[152] Kang T., Zhu Q., Jiang D., Feng X., Feng J., Jiang T., Yao J., Jing Y., Song Q., Jiang X. (2016). Synergistic targeting tenascin C and neuropilin-1 for specific penetration of nanoparticles for anti-glioblastoma treatment. Biomaterials.

[153] Lingasamy P., Tobi A., Kurm K., Kopanchuk S., Sudakov A., Salumäe M., Rätsep T., Asser T., Bjerkvig R., Teesalu T. (2020). Tumor-penetrating peptide for systemic targeting of Tenascin-C. Sci. Rep..

[154] Li S., Dong L., Pan Z., Yang G. (2023). Targeting the neural stem cells in subventricular zone for the treatment of glioblastoma: An update from preclinical evidence to clinical interventions. Stem Cell Res. Ther..

[155] Zhang G.-L., Wang C.-F., Qian C., Ji Y.-X., Wang Y.-Z. (2021). Role and mechanism of neural stem cells of the subventricular zone in glioblastoma. World J. Stem Cells.

[156] Matarredona E.R., Pastor A.M. (2019). Neural stem cells of the subventricular zone as the origin of human glioblastoma stem cells. Therapeutic implications. Front. Oncol..

[157] Huang T., Lu C., Zhang Y., Lin B.-y., Zhang Z.-j., Zhu D., Wang L., Lu Y. (2023). Effect of activating cancer-associated fibroblasts biomarker TNC on immune cell infiltration and prognosis in breast cancer. Ann. Med..

[158] Brösicke N., Faissner A. (2015). Role of tenascins in the ECM of gliomas. Cell Adhes. Migr..

[159] Cifola I., Fratini F., Cardinali B., Palmieri V., Gatti G., Selmi T., Donzelli S., Sacconi A., Cesarini V., Marei H.E. (2022). miRNome and proteome profiling of small extracellular vesicles secreted by human glioblastoma cell lines and primary cancer stem cells. Biomedicines.

[160] Du W., Xia X., Hu F., Yu J. (2024). Extracellular matrix remodeling in the tumor immunity. Front. Immunol..

[161] Kümper M., Steinkamp J., Zigrino P. (2022). Metalloproteinases in dermal homeostasis. Am. J. Physiol.-Cell Physiol..

[162] Angel I., Pilo Kerman O., Rousso-Noori L., Friedmann-Morvinski D. (2020). Tenascin C promotes cancer cell plasticity in mesenchymal glioblastoma. Oncogene.

[163] Kunikowska J., Morgenstern A., Pełka K., Bruchertseifer F., Królicki L. (2022). Targeted alpha therapy for glioblastoma. Front. Med..

[164] Dhaouadi S., Bouhaouala-Zahar B., Orend G. (2024). Tenascin-C targeting strategies in cancer. Matrix Biol..

[165] Dutoit V., Migliorini D., Ranzanici G., Marinari E., Widmer V., Lobrinus J.A., Momjian S., Costello J., Walker P.R., Okada H. (2018). Antigenic expression and spontaneous immune responses support the use of a selected peptide set from the IMA950 glioblastoma vaccine for immunotherapy of grade II and III glioma. Oncoimmunology.

[166] Grabowska M., Grześkowiak B.F., Szutkowski K., Wawrzyniak D., Głodowicz P., Barciszewski J., Jurga S., Rolle K., Mrówczyński R. (2019). Nano-mediated delivery of double-stranded RNA for gene therapy of glioblastoma multiforme. PLoS ONE.

[167] Bosiacki M., Gąssowska-Dobrowolska M., Kojder K., Fabiańska M., Jeżewski D., Gutowska I., Lubkowska A. (2019). Perineuronal Nets and Their Role in Synaptic Homeostasis. Int. J. Mol. Sci..

[168] Yong Y., Cai Y., Lin J., Ma L., Han H., Li F. (2024). Advancement in modulation of brain extracellular space and unlocking its potential for intervention of neurological diseases. Med-X.

[169] Wei R., Zhou J., Bui B., Liu X. (2024). Glioma actively orchestrate a self-advantageous extracellular matrix to promote recurrence and progression. BMC Cancer.

[170] Wang S., Zhang K., Huang Q., Meng F., Deng S. (2024). TLR4 signalling in ischemia/reperfusion injury: A promising target for linking inflammation, oxidative stress and programmed cell death to improve organ transplantation outcomes. Front. Immunol..

[171] Duan T., Du Y., Xing C., Wang H.Y., Wang R.-F. (2022). Toll-like receptor signaling and its role in cell-mediated immunity. Front. Immunol..

[172] Marzeda A.M., Schwenzer A., Didov B.S., Woolcock K., Richard J.-B., Jennings L.K., Julé A.M., Yang N., Davidson S., Sansom S. (2025). Investigating endogenous immune-mediated monocyte memory in rheumatoid arthritis. Ann. Rheum. Dis..

[173] Stierschneider A., Wiesner C. (2023). Shedding light on the molecular and regulatory mechanisms of TLR4 signaling in endothelial cells under physiological and inflamed conditions. Front. Immunol..

[174] Kim Y., de la Motte C.A. (2020). The role of hyaluronan treatment in intestinal innate host defense. Front. Immunol..

[175] You N., Chu S., Cai B., Gao Y., Hui M., Zhu J., Wang M. (2021). Bioactive hyaluronic acid fragments inhibit lipopolysaccharide-induced inflammatory responses via the Toll-like receptor 4 signaling pathway. Front. Med..

[176] Wang L., Wang H., Luo J., Xie T., Mor G., Liao A. (2022). Decorin promotes decidual M1-like macrophage polarization via mitochondrial dysfunction resulting in recurrent pregnancy loss. Theranostics.

[177] Wang S., Qu Y., Fang X., Ding Q., Zhao H., Yu X., Xu T., Lu R., Jing S., Liu C. (2023). Decorin: A potential therapeutic candidate for ligamentum flavum hypertrophy by antagonizing TGF-β1. Exp. Mol. Med..

[178] Ma C., Li Y., Li M., Lv C., Tian Y. (2025). Targeting immune checkpoints on myeloid cells: Current status and future directions. Cancer Immunol. Immunother..

[179] Xiao Z., Wang J., Yang J., Guo F., Zhang L., Zhang L. (2025). Dendritic cells instruct T cell anti-tumor immunity and immunotherapy response. Innov. Med..

[180] Tang M., Diao J., Gu H., Khatri I., Zhao J., Cattral M.S. (2015). Toll-like receptor 2 activation promotes tumor dendritic cell dysfunction by regulating IL-6 and IL-10 receptor signaling. Cell Rep..

[181] Galluzzi L., Vitale I., Warren S., Adjemian S., Agostinis P., Martinez A.B., Chan T.A., Coukos G., Demaria S., Deutsch E. (2020). Consensus guidelines for the definition, detection and interpretation of immunogenic cell death. J. Immunother. Cancer.

[182] Boaru D.L., Fraile-Martinez O., De Castro-Martinez P., Ortega M.A., Garcia-Montero C. (2026). What Is—And What Is Not—Immunogenic Cell Death? Functional Definitions, Experimental Standards, and Common Pitfalls. Int. J. Mol. Sci..

[183] Catanzaro E., Beltrán-Visiedo M., Galluzzi L., Krysko D.V. (2025). Immunogenicity of cell death and cancer immunotherapy with immune checkpoint inhibitors. Cell. Mol. Immunol..

[184] Galluzzi L., Kepp O., Hett E., Kroemer G., Marincola F.M. (2023). Immunogenic cell death in cancer: Concept and therapeutic implications. J. Transl. Med..

[185] Frevert C.W., Felgenhauer J., Wygrecka M., Nastase M.V., Schaefer L. (2018). Danger-associated molecular patterns derived from the extracellular matrix provide temporal control of innate immunity. J. Histochem. Cytochem..

[186] Halari C.D., Zheng M., Lala P.K. (2021). Roles of two small leucine-rich proteoglycans decorin and biglycan in pregnancy and pregnancy-associated diseases. Int. J. Mol. Sci..

[187] López-Cánovas A.E., Victoria-Sanes M., Martínez-Hernández G.B., López-Gómez A. (2025). Methods for determining the high molecular weight of hyaluronic acid: A review. Polymers.

[188] Pandey A., Kulshrestha R., Bansal S.K. (2021). Dynamic role of LMW-hyaluronan fragments and Toll-like receptors 2, 4 in progression of bleomycin induced lung parenchymal injury to fibrosis. Egypt. J. Bronchol..

[189] Hoarau A., Polette M., Coraux C. (2022). Lung hyaluronasome: Involvement of low molecular weight Ha (Lmw-Ha) in innate immunity. Biomolecules.

[190] Zuliani-Alvarez L., Marzeda A.M., Deligne C., Schwenzer A., McCann F.E., Marsden B.D., Piccinini A.M., Midwood K.S. (2017). Mapping tenascin-C interaction with toll-like receptor 4 reveals a new subset of endogenous inflammatory triggers. Nat. Commun..

[191] Yuan F., Yang Y., Zhou H., Quan J., Liu C., Wang Y., Zhang Y., Yu X. (2022). Heparanase in cancer progression: Structure, substrate recognition and therapeutic potential. Front. Chem..

